# Supplementary material for: Assessing the impact of artistic and cultural activities on the health and well-being of forcibly displaced people using participatory action research
Source: BMJ Open. 2019 Feb 20;9(2):e025465. doi: 10.1136/bmjopen-2018-025465 (PMC6398658; doi:10.1136/bmjopen-2018-025465)
Supplement: Supplementary file 1 [file bmjopen-2018-025465.draft_revisions.pdf]

BMJ Open is committed to open peer review. As part of this commitment we make the peer review history of every article we publish publicly available.

When an article is published we post the peer reviewers' comments and the authors' responses online. We also post the versions of the paper that were used during peer review. These are the versions that the peer review comments apply to.

The versions of the paper that follow are the versions that were submitted during the peer review process. They are not the versions of record or the final published versions. They should not be cited or distributed as the published version of this manuscript.

BMJ Open is an open access journal and the full, final, typeset and author-corrected version of record of the manuscript is available on our site with no access controls, subscription charges or pay-per-view fees (http://bmjopen.bmj.com).

If you have any questions on BMJ Open's open peer review process please email info.bmjopen@bmj.com

BMJ Open

Assessing the impact of artistic and cultural activities on the health and wellbeing of forcibly displaced people using participatory action research

Journal:	*BMJ Open*
Manuscript ID	bmjopen-2018-025465
Article Type:	Research
Date Submitted by the Author:	18-Jul-2018
Complete List of Authors:	Thomson, Linda; University College London Division of Biosciences<br>Clini, Clelia; Loughborough University - London, Institute for Media and Creative Industries<br>Chatterjee, Helen; University College London, Division of Biosciences
Keywords:	creative activities, forced displacement, participatory action research, post-traumatic stress disorder, refugees and asylum seekers

SCHOLARONE™
Manuscripts

For peer review only

Assessing the impact of artistic and cultural activities on the health and wellbeing of forcibly displaced people using participatory action research

Clelia Clini, Linda J Thomson, Helen J Chatterjee

Correspondence to: Helen J Chatterjee, UCL Division of Biosciences, 507B Darwin Building, University College London, London, WC1E 6BT, UK. email h.chatterjee@ucl.ac.uk telephone +44(0)2031084104 fax +44(0)2076797193

Clelia Clini, Institute for Media and Creative Industries, Loughborough University London, UK

Linda J Thomson, UCL Division of Biosciences, University College London, London, UK

Key words: creative activities; forced displacement; participatory action research; post-traumatic stress disorder; refugees and asylum seekers;

Word count: 4131

Abstract

Objective: Drawing upon a growing body of research suggesting that taking part in artistic and cultural activities benefits human health and wellbeing,[1-8] the objective was to develop a participatory action research (PAR) method for assessing the impact of arts interventions on forcibly displaced people. Although creative activities are seen to provide major benefits for refugees and asylum seekers, researchers have become 'increasingly conscious of the value of recording and analysing what has been happening'.[9 p1] Many European organisations provide creative activities aiming to reduce social isolation, as displaced people often display depression, anxiety, post-traumatic stress disorder and non-affective psychosis.[10-12]

Design: A study was conducted following PAR principles comprising observation, focus groups and in-depth semi-structured interviews.

Setting. London-based charity working with asylum seekers and refugees.

Participants: Refugees/asylum seekers (n=7), volunteers (n=7) and charity staff (n=8).

Results: Qualitative data was analysed using NVivo 11. Focus group findings showed that participants articulated reflections on the impact of creative activities around three main

For peer review only - http://bmjopen.bmj.com/site/about/guidelines.xhtml

themes: skills, social engagement, and personal emotions which were explored during interviews. Findings showed that artistic and cultural activities impacted positively on participants by helping them to find a voice, create a support network, and learn practical skills useful in the labour market.

Conclusions: The study expanded on arts and wellbeing research by exploring effects of cultural and creative activities on the psychosocial wellbeing of refugees and asylum seekers. By focusing on the relationship between arts, wellbeing and forced displacement, the study was instrumental in actively trying to change the narrative surrounding refugees and asylum seekers,[45] often depicted in negative terms in the public sphere.

Strengths and limitations of this study:

- Focus on collaborative research
- Different cultural and artistic activities studied
- Researcher spent five months in research setting
- Approximately twice as many females participated
- Focus groups attracted relatively low numbers

Introduction:

The recent All-Party Parliamentary Group on Arts Health and Wellbeing Inquiry Report shows a growing acknowledgement of the impact of arts and creative practices, stating 'arts engagement has a beneficial effect upon health and wellbeing and therefore has a vital part to play in the public health arena'.[13 p11] More than 70 years ago, the World Health Organisation defined health as a 'state of complete physical, mental and social wellbeing and not merely the absence of disease or infirmity'.[14 p2] As a key element of health, the notion of achieving increased wellbeing is central to many studies aimed at improving quality of life.[15 p5] Currently, consensus among practitioners defines wellbeing as 'the dynamic process that gives people a sense of how their lives are going through the interaction between their circumstances, activities and psychological resources or mental capital'.[16 p3] Rather than depending on a single factor, wellbeing is seen as the result of interplay between different elements, distinguishing personal wellbeing (emotional wellbeing, satisfying life, vitality, resilience, self-esteem and positive functioning) from social wellbeing (supporting relationships, trust and belonging).[17] Other authors emphasise 'resilience and flourishing, rather than just surviving',[18 p243] a crucial point when investigating the situation of

For peer review only - http://bmjopen.bmj.com/site/about/guidelines.xhtml

For peer review only

forcibly displaced people who often survive in extreme conditions and whose experience is often discussed in terms of dehumanisation.[19,20]

Research conducted in the field of forced displacement and mental health shows that 'asylum seekers and displaced people report high rates of pre-migration trauma',[20 p275] and significant levels of post-traumatic stress disorder (PTSD), depression and anxiety.[10, 12, 21-23] In addition to pre-migration trauma, refugees and asylum seekers often encounter post-migration living difficulties, such as socio-economic disadvantage, employment barriers, social and emotional isolation, racism and hostility, experience of detention, and uncertainty related to the asylum application process.[10, 12, 21, 24] If, as authors suggest, there is a strong link between social and economic conditions in terms of income, employability, housing and mental health,[25-29] then asylum seekers and refugees are at greater risk of developing mental health issues than the general population.

Arts-and-health practitioners believe that 'aesthetics act upon our senses to make us feel more, hear more and see more than we otherwise might… feelings are intertwined with mental, physical, spiritual and social health'.[30 p4] Several studies explored the relationship between arts, health and wellbeing and a growing body of evidence[13] suggests that cultural participation (including music, art making, theatre, dance, museum and heritage activities) enhances human health and wellbeing.[1-8, 31] Research conducted in museums-and-health, for example, suggests that 'museum and art gallery encounters can help with a range of health issues, enhance wellbeing, and build social capital and resilience'[3 p286]. According to research conducted in Norway involving over 50,000 adults,[32] participation in both receptive/passive and creative/active cultural activities was significantly associated with good health, life satisfaction, and low anxiety and depression. Findings demonstrated how creative practices, such as knitting, had a positive impact on people suffering from depression and post-traumatic stress disorder because 'the movements involved in knitting are bilateral, rhythmic, repetitive, and automatic'.[32 p40] The authors attributed the positive mood of knitters to enhanced production of serotonin resulting from repetitive movements and that bilateral processes appeared to engage brain capacity and facilitate a meditative-like state more readily than unilateral ones.

The current study was conducted in the light of mental health and arts/museums-and-health evidence given the large number of organisations that engage migrants, refugees and asylum seekers in the arts, with nearly 200 of these in the UK.[9] The current research was conducted at one such organisation, Helen Bamber Foundation (HBF), a charity offering support to refugees and asylum seekers as victims of torture and human rights violations.

For peer review only - http://bmjopen.bmj.com/site/about/guidelines.xhtml

HBF adopts a holistic approach to support its clients that includes cultural and creative activities within a model of integrated care. The model is based on the needs of traumatised asylum seekers and refugees who often require simultaneous assistance on a variety of fronts (e.g. psychological support might not be effective if a person does not have a place to sleep or lives in a dangerous situation, though giving people a place to sleep is not enough to overcome trauma). Their model encompasses services such as legal and health support (psychological and physical), welfare and housing advice, and a creative arts programme (CAP) including art, photography, knitting and textiles, singing, English and computing, led by professional volunteers and attended by nearly 100 clients. By focusing on the relationship between arts, wellbeing and forced displacement, the study aimed to determine why participation in creative arts enhanced the sense of wellbeing experienced by HBF clients, and contribute to the current debate on migration and public health.

Method

Design

Qualitative research conducted comprised participant observation, focus groups and one-to-one semi-structured interviews.[33] The research was operationalised according to the principles of participatory action research (PAR) as a 'way of opening up space for dialogue and conversation',[34 p28] aiming 'understand and also improve a particular situation',[35 p1] The approach encourages an active contribution in the production of knowledge within a collaborative framework, emphasising 'equal partnerships',[36 p167] and the 'role of the participant in the design, implementation, and dissemination of the research'.[37 p78] The collaborative character of PAR is grounded in efforts to 'democratise the research process',[35 p3] so it is valuable for everyone involved and where 'equal weight and consideration is given to the contributions of both the community and academic partners'[36 p170]. By offering equal weight to contributions, participants develop a sense of ownership, which in turns fuels the feeling of empowerment, one of the pillars of PAR, especially when community-based.[39]

Participants

The research recruited an opportunity sample of participants who, in keeping with PAR were called 'co-researchers' and who had differing involvement in the project depending on their role at HBF (clients, volunteers or staff). Information leaflets and researcher contact details

For peer review only - http://bmjopen.bmj.com/site/about/guidelines.xhtml

were placed in reception to encourage participation and clients were recruited directly by the researcher while based at HBF. The criteria for recruitment were that clients had received therapy for at least two years prior to the research and, while still vulnerable, had entered the integration stage of the model of integrated care, and that they attended at least one of the five CAP groups (art, knitting, photography, singing and textiles). Volunteers and staff were recruited via email and personal contact. Co-researchers (n=22: 7m) participated in focus groups comprising clients (n=6), volunteers (n=4) and staff (n=8), and interviews (n=18) comprising clients (n=7), volunteers (n=7) and staff (n=4).

Patient and public involvement

The development of the research questions and outcome measures about the ffects of cultural and creative activities on the psychosocial wellbeing of refugees/asylum seekers was informed by HFB clients, volunteers and staff taking part in PAR. Focus groups and interviews gave voice to participants' priorities, experience and preferences but, due to the vulnerability of clients, no personal information was requested as the process of remembering could have been difficult for PTSD sufferers, [10, 22] and although some participants mentioned personal issues and experiences, they were not encouraged to do so. Participants were involved as co-researchers within the research collaboration and data generated by their involvement informed focus groups and interviews. Participants were indirectly involved in the recruitment process by recommending that other people join them, though the researcher needed to ensure they matched recruitment criteria (above). Participants were directly involved in conducting the study and determining the interview questions. Results were disseminated to participants through an end-of-project event and on-going communication with the researcher, and an internal report will be lodged at HBF.

Data Collection

Data collection carried out by the location-based researcher consisted of three stages: i) participant observation (months 1-2), ii) focus groups (months 3-4) and iii) semi-structured interviews (month 5). Participant observation was used as a starting point 'for studying how organisations work, the roles played by different staff and the interaction between staff and clients'.[33 p32] In stage i) the researcher attended creative classes for clients and spent a day a week working from HBF (12-15 hours per week) to determine the nature of focus groups. In stage ii) four focus groups were held, the first and final with clients from arts, photography, singing, and textiles, the second with volunteers, and the third with staff. In the

For peer review only - http://bmjopen.bmj.com/site/about/guidelines.xhtml

focus groups, participants were asked to discuss research questions (Table 1) involving ways of gathering information on the impact of creative activities, and reasons for attending groups (or in the case of volunteers and staff, reasons for involvement). All focus groups were organised informally to facilitate development of discussion among participants and allow an exchange of experiences and ideas. Ideas which emerged during focus groups laid the basis for the first drafts of interview questions, which were tailored to clients, volunteers or staff (Table 2). In line with the collaborative ethos, drafts were circulated to receive feedback from co-researchers prior to the interview stage, and volunteers and staff were consulted and asked to provide comments and suggestions on the questions. For stage iii) interviews carried out with were semi-structured to allow free expression within the research constraints and limit potential influence of the researcher.[33,34]

INSERT TABLE 1 ABOUT HERE

INSERT TABLE 2 ABOUT HERE

Data analysis

A first informal thematic analysis was conducted by the HBF-based researcher while writing field notes consisting of a chronicle of descriptive rather than analytic events to provide the ‘raw material of the research’.[33 p38] The process of transcribing notes was used to identify key points connected to the research question. This preliminary analysis informed the focus group topics where themes were discussed more in detail. After the first round of focus groups, all notes were compiled into a Word document and repeatedly read in search of recurring subjects and themes. Thematic analysis of focus group outcomes were explored in detail during interviews. All interviews were recorded and transcribed to become familiar with the data gathered and begin the coding process [40]. In the transcript, all details were recorded verbatim (e.g. sighs, laughter, silences, and tears) as their exclusion could have changed the meaning expressed.[41] Transcriptions were uploaded into NVivo11 to produce nodes from coding-relevant concepts. NVivo’s word frequency query facilitated the search for key words (including stem words and synonyms) grouped together under the same theme. The text search query provided a comprehensive analysis of data. After coding of data into initial themes,[40] a review of themes followed to refine the analysis.

For peer review only - http://bmjopen.bmj.com/site/about/guidelines.xhtml

Results

Focus Groups

Thematic analysis of focus group data showed consistency in the way all HBF participants (clients, volunteers and staff) articulated their reflections on the impact of creative activities. In discussing the value of engaging in creative arts, participants highlighted the benefits of activities as:

- Learning new practical skills or improving those previously learnt;
- Acquisition of technical, language, social and life skills;
- Development of new social relationships based on respect and mutual recognition;
- Opportunities to meet people, forge friendships, counter loneliness and develop a sense of belonging in a safe space;
- Value of peer-learning and mutual support;
- Improvement in mood and self-confidence; and
- Freedom of expression and developing a new identity other than refugee/asylum seeker.

Thematic clusters emerged from around three main overarching (and overlapping) themes from these benefits: 'social aspects-friendships', 'skills', and 'mood-personal sphere'; the latter was sub-divided into 'brain' (how creative activities helped the brain), 'routine', self-expression' and 'confidence'.

Interviews

During the interviews, respondents tended elaborated on skills, both practical and social, and also commented on the effect that attendance had on their mood and emotion.

Learning new practical skills: One of the themes which emerged strongly during interviews was that creative arts groups allowed participants to learn new skills and/or to improve prior skills. Skill improvement was important for asylum seekers without the right to work. As a client pointed out:

I have learned a lot of skills: I've learned sewing, now I'm learning art, I can do better, before I didn't know how to draw a person, to mix colours, you know such things... so you keep on learning, slowly by slowly, then in the end you find yourself,

For peer review only - http://bmjopen.bmj.com/site/about/guidelines.xhtml

you're a professional, so that is very great... to me... I like to come, always and attend, and listen to what they tell me, and I do it.

Or, as another client explained:

I've learned new skills [...] and you can never waste your time learning new skills. New skills always help you in your life, always. So, everything I've learned will help me. You think like "film club, learning how to edit, etc.". People pay people on YouTube to edit their films, and I'm, like, I could just do my own, if I wanted to have a YouTube channel, I can do my own.

The improvement of skills was a benefit that provided a sense of achievement and improved self-confidence, illustrating the overlapping nature of themes that emerged. Talking about new skills learnt, one client observed:

When you are in this situation it feels like life has, in a way, stopped. And you can't do anything to change it. But by doing all this activity you feel, or I felt…it was like, you know, that I was learning something in my life, rather than just waiting. […] For instance, if I go for a job somewhere, where you have to write what skills you have, so I could include all of this. I mean I don't have a certificate or, like, proper qualifications, but I've learned all those skills, so I feel like, it is, in a way, impressive? Because you feel like, you know, rather than just waiting and not doing anything, you have been learning.

Self-improvement was seen not only as a result of learning from professionals, but also from peer-learning; several clients commented on the fact that they learnt from friends as well as volunteers, pointing to the importance of social aspects of the classes.

Acquisition of technical, language, social and life skills: One of the benefits of being engaged in creative activities that emerged was that participants had the opportunity to overcome social isolation and create a social network. As one client pointed out:

The groups changed me because you know when I come, I feel lonely and doesn't have anybody, I was lonely I don't even know whether there will be a home from morning to live in. But when I started coming these groups, I told you I feel active, I feel coming to see people, that I speak to, I don't even know how to communicate with people before. I told you because of my coming here I now feel to communicate to people. Before I never communicate to people, I don't know how to do it. And I don't know how to play with people. But these groups make me now taught me: play with people, to meet me.

For peer review only - http://bmjopen.bmj.com/site/about/guidelines.xhtml

The social aspect was an important point reiterated in all interviews; fellow clients were described as ‘friends’ but also as ‘brothers and sisters’. Arts activities allowed clients to create a community characterised by solidarity, as one client explained:

If you're in a situation where you've been completely isolated from people for a while and you just don't know who to trust, or to be around people, it's one of those spaces where you can get to meet people, socialise, and actually make friends. […] And these friendships do last. A life-time, because you understand what the person has gone through, you don't have to explain a lot, you know, they just know that you've been through something terrible. […] So, it's easier when you have people here who just understand what you've possibly been through.

This remark connects with the observation that ‘engagement in participative creative arts activities in communities can help to build social capital, address loneliness and social isolation, and build personal confidence and a sense of empowerment’.[25 p32] The awareness of experiencing similar situations makes clients feel free to share their own experiences with one another but also not to, if they do not feel like opening up:

There is a sense of community: you know you go there, you know there are people like you in the same situation you're not going to be judged, so it's that sense of community: we know what's going on, we don't have to talk about it. […] It's a distraction from immigration and we know we are all going through it, but we don't have to talk about it. There are other things going on in life, and we talked about, for example in the art group, the works we produced and what we could do, what we could achieve and get inspired by each other's work.

Again, this remark introduced the third broad theme which emerged out of the interview analysis, which was the impact of creative arts on personal mood and emotion.

Mood and Emotion: Learning or improving skills while forging long-lasting relationships in a context deep social isolation (outside of HBF), inevitably has a substantial impact on mood and emotion. In discussing this aspect of creative arts, interviewees focused particularly on how groups allowed them to have a routine, a ‘luxury’ a client said ‘when you can’t do nothing but waiting for the government to decide on your right to stay in the country’, and this routine combined with skills learning/improving was thought to have a positive impact on their mental health condition. ‘Having something to do’ and ‘something to look forward to’ were recurrent expressions during interviews. As one client noted:

For peer review only - http://bmjopen.bmj.com/site/about/guidelines.xhtml

I used to come here every day of my life: Monday, Tuesday, Wednesday...because I don't have anywhere to go, so I just come here, and I attached myself to the groups, we met, we talk, we...then I've gone to knit, I've gone to do computer, reading, many things I learnt here, so I just like it.

Another client elaborated further:

It's something that I look forward to. One thing you have to bear in mind is that I am not working, and I am not studying, you know, so it was the only thing that I would look forward to because it was something to do, otherwise I would just be at home, doing nothing, you know, feeling very sorry for myself, getting upset all the time. I mean I still feel that way but at least there's something to look forward to, when you don't have hope or something to look forward to, it increases your depression levels, so for me it's helped me greatly.

The benefits of having a routine were increased because it involved creative activities:

There is nothing else for you and it's also a way of, like, using your time wisely, 'cause at the end of the day most of the time you're just sitting down doing nothing, and doing nothing slowly begins to affect your mind, your brain, you know you become lazy, you know you literally are just in a four-squared room […]

According to clients and volunteers, creative activities had a positive impact on mood because they allowed people to exercise their brain and find a voice to express themselves. As a client stated:

[Attending creative groups] gives you a lot of confidence, you know. And it allows your brain to think outside of the box because I think we are just in that box and... but when you attend these classes, your mind, you know, opened and you learn new things and you want to then learn other things as well.

This reflection highlights the important link between arts and feelings; as another client said:

It is more of expressing, more of letting go, it's like getting a spirit out of you, and you don't have necessarily have to tell someone "I've done this because of that and that, and this", you know. Because sometimes you just don't find the voice to talk about it and, I, as a person am really shy and, you know, I feel easily embarrassed, you know. […] So... that's why I'm into arts, yeas I'm doing arts really.

Clients agreed that the possibility of expressing themselves without having to articulate their feelings, helped them grow emotionally and gain confidence. It was not only clients who benefited from CAP; research findings also pointed to the beneficial impact of CAP on volunteers, a point stressed by all volunteers when discussing personal experiences.

For peer review only - http://bmjopen.bmj.com/site/about/guidelines.xhtml

Discussion

Creative and cultural activities carried out over a sustained period of five months provided refugees and asylum seekers with new skills, that included practical and technical skills, and social and life skills involving language acquisition obtained partly through informal peer learning and mutual support. Learning new skills contributed to the sense of wellbeing and empowerment experiences by HBF clients. Furthermore, activities appeared to positively enhance mood and emotion both for clients and volunteers. The time spent by the researcher at the HBF was vital to become familiar with the context, get to know people involved in the organisation, and explain the research to make it possible to recruit co-researchers from among clients, volunteers and staff for focus groups and interviews. Moreover, the fact that several clients often attended more than one group a week (in certain cases all five groups) made it possible to forge trusting relationships and recruit co-researchers, allowing them to feel comfortable in speaking their mind regarding the research.

In keeping with previous research, creative and cultural participation lead to improved social health [30] and aligned with a growing body of evidence[13] to suggest that the five selected CAP activities enhanced mental wellbeing[1-8, 31] helping clients to develop self-confidence and resilience.[3] A possible issue here was that the number of groups attended by clients, that varied from one to three throughout the week, was not taken into account, so it is not evident as to whether increased participation would have enhanced positive aspects of wellbeing at a higher or faster level. Although passive participation was not compared,[32] active and creative participation in the CAP groups specifically benefited refugees and asylum seekers, as their status in the UK would not have permitted alternative occupation or employment, so may not have left their homes and met other people on a regular basis.

In terms of study weaknesses, the greater number of female participants (approximately twice that of males) was due to the fact that most creative arts groups were led by female volunteers (only two of ten volunteers were male) and most participants in these groups were also female except for the singing group, where numbers were roughly equal. Consequently, it was easier for the female researcher to forge relationships with women rather than with men. With the singing group, while one male choir member took part in one focus group, it was difficult to engage male participants in the research; given the nature of the activity, conversation was limited to the first minutes before arrival as once rehearsals started, it became difficult to forge meaningful relationships with participants.

A further weakness was the low level of attendance for focus groups. As a result of undergoing difficult situations, clients in particular had circumstances that could change

For peer review only - http://bmjopen.bmj.com/site/about/guidelines.xhtml

quickly, consequently, not all of those who expressed an interest in participating were able to do so but most were nevertheless happy to informally discuss the topics debated afterwards; other clients expressed an interest in being kept in the loop regarding the progress of the research. As for volunteers, while at least one volunteer per group was involved in the research at any point, it was difficult to organise focus group times convenient for everyone, so again themes and research methods were discussed in more informal ways with the researcher based at HBF during weekly meetings with clients.

Conclusions

This study expanded on arts and wellbeing research by exploring the effects of cultural and creative activities on the psychosocial wellbeing of refugees and asylum seekers. The activities at HBF provided opportunities for clients to meet people and develop new social relationships based on respect and mutual recognition. In turn, these opportunities countered loneliness and allowed refugees and asylum seekers to develop a sense of belonging in a safe space. The opportunities further supported freedom of expression and the possibility of developing a new identity to counter the negative stereotype of being viewed as a refugee or asylum seeker, leading to sustainable improvements in positive mood and self-confidence, countering anxiety and depression, and other mental health issues. By focusing on the relationship between arts, wellbeing and forced displacement and in keeping with PAR, the study has been instrumental in actively trying to change the narrative surrounding refugees and asylum seekers,[42] often depicted in negative terms in the public sphere. Refugee and asylum seeker organisations should consider commissioning PAR to involve their clients, volunteers and staff in appraising and improving the current provision for migrants to the UK, particularly in terms of broad-ranging skill sets and psychosocial support.

Contributors

CC, LT and HC made substantial contributions to the conception and design of the work; drafting and critically revising the work for intellectual content; and the final approval of the version published. CC conducted the acquisition, analysis and interpretation of data for the work. All authors agree to be accountable for all aspects of the work. We would like to thank the clients, volunteers and staff who were co-researchers in the project.

For peer review only - http://bmjopen.bmj.com/site/about/guidelines.xhtml

Funding

The work was supported by the Global Challenges Research Fund (GCRF) Grant reference: ES/P003818/1 PI: HC

Ethics

The study gained approval from UCL Ethics Committee (Ethics Application 4526/002 Co-developing a method for assessing the psychosocial impact of cultural interventions with displaced people: Towards an integrated care framework) to carry out the research with potentially vulnerable participants.

Competing interests

The authors have no competing interests.

Data sharing statement

Due to the confidential nature of the human participant data, it will not be freely available via free access databases. Any request for data should be addressed to the corresponding author.

References

1. Bygren LO, Johansson S, Konlaan BB, Grjibovski AM, Wilkinson AV, Sjöström M. Attending cultural events and cancer mortality: A Swedish cohort study. Arts Health: Int J Res Pol Pract 2009;1:64–73. doi:10.1080/17533010802528058
2. Camic P, Chatterjee H. Museums and art galleries as partners for public health interventions. Perspect Public Health 2013;133:66–77.
3. Chatterjee H. Museums and art galleries as settings for public health interventions. In S Clift, P Camic (Eds.), Oxford Textbook of Creative Arts, Health, and Wellbeing: International Perspectives on Practice, Policy and Research. New York, Oxford: Oxford University Press 2016; p.281–289.
4. Clift S, Skingley A, Coulton S, Rodriguez J. The effectiveness and cost-effectiveness of a participative community singing programme as a health promotion initiative for older people: protocol for a randomised controlled trial. BMC Public Health 2011;11:1–6. doi:10.1186/1471-2458-11-142
5. Clift S, Camic P. Oxford Textbook of Creative Arts, Health, and Wellbeing: International Perspectives on Practice, Policy and Research. New York, Oxford: Oxford University Press, 2016.

For peer review only - http://bmjopen.bmj.com/site/about/guidelines.xhtml

6. Konlaan BB, Bygren LO, Johansson S. Visiting the cinema, concerts, museums or art exhibitions as determinant of survival: a Swedish fourteen-year cohort follow-up. Scand J Public Health 2000;28:174–178. doi:10.1177/14034948000280030501
7. Staricoff RL. Arts in health: A review of the medical literature. London: Arts Council England, Research Report 2004;36:1–91. http://www.artsandhealth.ie/wp-content/uploads/2011/08/AHReview-of-Medical-Literature1.pdf
8. Staricoff RL. Arts in health: The value of evaluation. Perspect Public Health 2006;126:116–120. http://journals.sagepub.com/doi/pdf/10.1177/1466424006064300
9. Kidd B, Zahir S, Khan S. Arts and Refugees: History, Impact and Future. London: Arts Council England, 2008. https://baringfoundation.org.uk/wp-content/uploads/2014/10/ArtsandRefugees.pdf
10. Katona C. Non-affective psychosis in refugees. Br Med J 2016;352:i1279. doi:10.1136/bmj.i1279
11. Hollander A, Dal H, Lewis G, Magnusson c, Kirkbride JB, Dale H, Dalman C. Refugee migration and risk of schizophrenia and other non-affective psychoses: cohort study of 1.3 million people in Sweden. Br Med J 2016;352:i1030. https://doi.org/10.1136/bmj.i1030
12. Robjant K, Robbins I, Senior V. Psychological distress amongst immigration detainees: A cross-sectional questionnaire study. Brit J Clin Psych 2009;48:275–286. doi:10.1348/014466508X397007
13. All-Party Parliamentary Group on Arts, Health and Wellbeing (APPGAHW). Creative Health: The Arts for Health and Wellbeing. Inquiry Report. 2017; http://www.artshealthandwellbeing.org.uk/appg-inquiry/
14. World Health Organization. The Preamble of the Constitution of the World Health Organization. Geneva: World Health Organization, 1946. https://www.scielosp.org/scielo.php?pid=S0042-96862002001200014&script=sci_arttext&tlng=pt
15. White S, Blackmore C. Cultures of Wellbeing: Method, Place, Policy. Basingstoke: Palgrave Macmillan, 2015.
16. New Economics Foundation (NEF). 2009. National accounts of wellbeing: What is wellbeing? NEF. http://www.nationalaccountsofwellbeing.org/learn/what-is-well-being.html
17. New Economic Foundation (NEF). National Accounts of Well-being: Bringing Real Wealth onto the Balance Sheet. 2009. https://neweconomics.org/uploads/files/2027fb05fed1554aea_uim6vd4c5.pdf

For peer review only - http://bmjopen.bmj.com/site/about/guidelines.xhtml

18. Ander E, Thomson LJ, Noble G, Lanceley A, Menon U, Chatterjee H. Generic well-being outcomes: towards a conceptual framework for well-being outcomes in museums. Museum Management Curatorship 2011; 26:237–259. doi:10.1080/09647775.2011.585798

19. Crawley H, Düvell F, Jones K, MCMahon s, Sigona, N. Destination Europe? Understanding the dynamics and drivers of Mediterranean migration in 2015. MEDMIG Final Report 2016. www.medmig.info/research-brief-destination-europe.pdf

20. Vaughan-Williams N. "We are *not* animals!" Humanitarian border security and zoopolitical spaces in Europe. Political Geography 2015;45:1–10. doi:10.1016/j.polgeo.2014.09.009

21. Alpak G, Unal A, Bulbul F, Sagaltici E, Bez Y, Altindag A et al. Post-traumatic stress disorder among Syrian refugees in Turkey: A cross-sectional study. Int J Psych Clinic Practice 2015;19:45–50. doi:10.3109/13651501.2014.961930

22. Schubert CC, Punamäki R. Mental health among torture survivors: cultural background, refugee status and gender. Nordic J Psych 2011;65:175–182. doi:10.3109/08039488.2010.514943

23. Silove D, Sinnerbrink I, Field A, Manicavasagar V, Steel Z. Anxiety, depression and PTSD in asylum-seekers: associations with pre-migration trauma and post-migration stressors. Brit J Psych 1997;170:351–357. doi:10.1192/bjp.170.4.351

24. Aragona M, Pucci D, Mazzetti M, Geraci S. Post-migration living difficulties as a significant risk factor for PTSD in immigrants: a primary care study. Italian J Public Health 2012;9:e7525;1–8. doi:10.2427/7525

25. Allen J, Allen M. The social determinants of health, empowerment and participation. In S Clift, P Camic (Eds.), Oxford Textbook of Creative Arts, Health, and Wellbeing: International Perspectives on Practice, Policy and Research. New York, Oxford: Oxford University Press; 2016, p27–34.

26. Fisher M. Why mental health is a political issue. The Guardian, July 16, 2012. https://www.theguardian.com/commentisfree/2012/jul/16/mental-health-political-issue

27. Griffin J. The Lonely Society. London: The Mental Health Foundation. https://www.mentalhealth.org.uk/sites/default/files/the_lonely_society_report.pdf

28. Marmot M, Allen J, Goldblatt P, Boyce T, McNeish D, Grady M et al. Fair Society, Healthy Lives: The Marmot Review. 2010; http://www.instituteofhealthequity.org/resources-reports/fair-society-healthy-lives-the-marmot-review/fair-society-healthy-lives-full-report-pdf

For peer review only - http://bmjopen.bmj.com/site/about/guidelines.xhtml

29. Paul K, Moser K. Unemployment impairs mental health: Meta-analyses. J Vocational Behavior 2009;74:264-282. doi:10.1016/j.jvb.2009.01.001
30. Prior RW. Editorial. J Applied Arts Health 2009;1:3–6.
31. Chatterjee HJ, Noble G. Museums, Health and Wellbeing. Farnham: Ashgate, 2013.
32. Cuypers K, Krokstad S, Holmen TL, Knudtsen MS, Bygren LO, Holmen J. Patterns of receptive and creative cultural activities and their association with perceived health, anxiety, depression and satisfaction with life among adults: the HUNT study, Norway. J Epidemiol Community Health 2012;66:698–703. https://jech.bmj.com/content/jech/66/8/698.full.pdf
33. Pope C, Mays N. Qualitative Research in Health Care, 3rd Edition. Oxford: Blackwell, 2006.
34. Kemmis S, McTaggart R, Nixon R. The Action Research Planner: Doing Critical Participatory Action Research. Singapore: Springer 2014.
35. Bradbury H. Introduction: How to Situate and Define Action Research. In H Bradbury (Ed). The Sagw Handbook of Action Research, London: Sage 2015. p.1–9.
36. Nicolaidis C, Raymaker DM. Community based participatory research with communities defined by race, ethnicity, and disability: Translating theory to practice. In H Bradbury (Ed.), The Sage Handbook of Action Research. London: Sage 2015. p.167–178.
37. Daykin N, Stickley T. The role of qualitative research in arts and health. In S Clift, P Camic (Eds.), Oxford Textbook of Creative Arts, Health, and Wellbeing: International Perspectives on Practice, Policy and Research. New York, Oxford: Oxford University Press 2016, p.73–82.
38. Vaughn LM, Jacquez F. Community-Based Participatory Research Studies Involving Immigrants. In S Coughlin, SA Smith, ME Fernandez (Eds.), Handbook of Community-Based Participatory Research. New York, Oxford: Oxford University Press, 2017. p.115–130.
39. Blumenthal DS. Is Community-based participatory research possible? Am J Prev Med. 2011;40:386–389.
40. Braun V, Clarke V. Using thematic analysis in psychology. Qual Res Psychol 2006;3:77–101.
41. Pope C, Ziebland S, Mays N. Analysing Qualitative Data. In C Pope, N Mays. Qualitative Research in Health Care, 3rd Edition. Oxford: Blackwell. 2006. p.63–81.
42. Khosravi S. 'Illegal' Traveller: An Auto-ethnography of Borders. Basingstoke: Palgrave MacMillan 2010.

For peer review only - http://bmjopen.bmj.com/site/about/guidelines.xhtml

Table 1. Focus group questions

Clients:
1. Why do you like the HBF creative arts programme?
2. How would you gather information or evidence about the benefits of artistic activities?
3. Which creative arts classes have a positive impact on the lives of refugees? Circle the option(s) that you think work best or add another one. Arts and crafts Drawing Films Knitting Photography Singing Textiles.
Volunteers:
1. Why do you volunteer at the HBF?
2. How do you think your group benefits clients?
3. How would you collect evidence on the effects of participating in artistic activities?
Staff:
1. Why do you recommend clients to attend Creative Arts groups?
2. What do you think are the benefits of attending arts groups?
3. How would you collect evidence on the effects of participating in artistic activities?

For peer review only

Table 2. Interview questions

Clients:

1. How long have you been attending the CAP classes for?
2. Which groups do you attend?
3. Why did you choose this/these groups(s)?
4. What do you like the most about these groups?
5. Is there anything about the groups that you find difficult?
6. Anything you would like to change?
7. Is there a group you prefer? Which one? Why?
8. How do you feel when you attend this group?
9. Do you think there are benefits of attending these groups? If so what are they?
10. During our previous meeting, it emerged that participants feel that groups allow them to learn new or improve already acquired skills. Do you agree? Have you learnt any new skill? And what do you think about the possibility of learning new skills? (How does that impact your life)
11. Another element which emerged during our meeting is that people enjoy the social aspect of these groups: meeting people and finding new friends. What do you think about this? [supporting questions: do you like being around other people? Why? What happens when you attend a session? Have you made new friends? How do you feel about that?]
12. Among all of the reasons why you enjoy attending the creative arts group, which one is the one that you feel strongest about?
13. Why do you keep attending?
14. If you could compare the way you were feeling before joining any of these activities and the way you feel now, would you say you feel any different? Explain
15. Creative activities of course are part of the support that HB offers to its clients. Do you think that you would feel the same about yourself today even without attending these creative activities groups? Why?
16. Do you think that taking part in these creative activities has had any influence on the way you see yourself? And the way you see yourself in London?
17. If you were to recommend someone to join a group, what would you say?

For peer review only - http://bmjopen.bmj.com/site/about/guidelines.xhtml

Volunteers:

1. What do you teach?
2. How long have you been volunteering for?
3. Why did you decide to volunteer?
4. How did you learn about the Helen Bamber Foundation?
5. What do you think your group offers clients?
6. What are the main challenges you face/have faced as a volunteer?
7. Do you find it difficult to engage clients in the activity you coordinate? What do you think clients like about the way you (and your colleagues) handle the sessions? Is there anything that clients don't like?
8. How would you describe a typical session?
9. What do you think people like the most about your group?
10. Do you ever discuss emotions with clients? Or their personal situation?
11. Look at the notion of wellbeing defined by NEF:

Wellbeing as 'the dynamic process that gives people a sense of how their lives are going through the interaction between their circumstances, activities and psychological resources or 'mental capital.' (NEF 2008 p3).

According to the NEF, there are different components to wellbeing, such as:
'Personal wellbeing (emotional wellbeing, satisfying life, vitality, resilience, self-esteem and positive functioning) and social wellbeing, including supporting relationships and trust and belonging.' (NEF 2009).

12. Do you think that the activity you lead has any impact on the wellbeing of clients? Elaborate
13. If you were asked to recruit people for your group, what would you say to convince them to join?
14. Would you use different words to promote your group with men and women? Why?
15. Do you think that your group makes a difference in the lives of clients? How? Why?
16. And what about your own life? What is the impact of your volunteer activity on your own life?

For peer review only - http://bmjopen.bmj.com/site/about/guidelines.xhtml

Staff:

1. How long have you been working at HB for?
2. Why did you decide to work here?
3. What is your role?
4. As part you work, do you have any direct contact with clients?
5. Could you say a few words on your relationship with clients?
6. How would you describe your own experience at HB so far?
7. What are the main challenges that you face/have faced in your position?
8. Do you think that your own gender influences the ways in which clients relate to you?
9. In your position, do you discuss clients' personal situations and feelings?
10. If so, do you think that the cultural background of clients affects the way in which you discuss emotions? If it does, how and why?
11. What are the main challenges of discussing emotions with clients?
12. Could you say a few words on the Model of Integrated Care? Do you think it works? And if so, why?
13. Do you recommend clients to attend any of the CAP groups? What do you say in this case?
14. What role do you think cultural and creative activities play in the recovery of clients?
15. Are there any particular activities that, in your opinion, are more popular with clients?
16. Would you say that the programme makes a difference in the lives of clients? If yes, please elaborate
17. Have you witnessed clients benefiting from their own participation in the creative arts programmes? If yes, in what ways?

For peer review only

For peer review only - http://bmjopen.bmj.com/site/about/guidelines.xhtml

Research and reporting methodology
Revised **Standards for Quality Improvement Reporting Excellence** (**SQUIRE 2.0**) publication guidelines

Notes to authors

- The SQUIRE guidelines provide a framework for reporting new knowledge about how to improve healthcare.
- The SQUIRE guidelines are intended for reports that describe system level work to improve the quality, safety and value of healthcare, and used methods to establish that observed outcomes were due to the intervention(s).
- A range of approaches exists for improving healthcare. SQUIRE may be adapted for reporting any of these.
- Authors should consider every SQUIRE item, but it may be inappropriate or unnecessary to include every SQUIRE element in a particular manuscript.
- The SQUIRE glossary contains definitions of many of the key words in SQUIRE.
- The explanation and elaboration document provides specific examples of well-written SQUIRE items and an in-depth explanation of each item.
- Please cite SQUIRE when it is used to write a manuscript.

Text section and item name	Page/line no(s). info is located
**Title and abstract**	
1. **Title**	1/1-2/1
Indicate that the manuscript concerns an initiative to improve healthcare (broadly defined to include the quality, safety, effectiveness, patient-centredness, timeliness, cost, efficiency and equity of healthcare).	
Manuscript concerns an initiative to improve healthcare for forcibly displaced people.	
2. **Abstract**	21/1-9/2
a. Provide adequate information to aid in searching and indexing.	
b. Summarise all key information from various sections of the text using the abstract format of the intended publication or a structured summary such as: background, local problem, methods, interventions, results, conclusions.	
The structure abstract summarises all key information under headings of: objective, design, setting, participants, results, and conclusions.	
**Introduction: Why did you start?**	
3. **Problem description** - Nature and significance of the local problem.	2/19-2/22
4. **Available knowledge** - Summary of what is currently known about the problem, including relevant previous studies.	2/22-2/14
5. **Rationale** - Informal or formal frameworks, models, concepts and/or theories used to explain the problem, any reasons or assumptions that were used to develop the intervention(s) and reasons why the intervention(s) was expected to work	3/3-3/29
6. **Specific aims** - Purpose of the project and of this report.	3/30-4/12
**Methods: What did you do?**	
7. **Context** - Contextual elements considered important at the outset of introducing the intervention(s).	4/17-4/29

For peer review only

8. **Intervention(s)**	
a. Description of the intervention(s) in sufficient detail that others could reproduce it.	5/26-6/11
b. Specifics of the team involved in the work.	4/32-5/8
9. **Study of the intervention(s)**	
a. Approach chosen for assessing the impact of the intervention(s).	4/17-4/23
b. Approach used to establish whether the observed outcomes were due to the intervention(s).	4/23-4/29
10. **Measures**	
a. Measures chosen for studying processes and outcomes of the intervention(s), including rationale for choosing them, their operational definitions and their validity and reliability.	5/27-5/29
b. Description of the approach to the ongoing assessment of contextual elements that contributed to the success, failure, efficiency and cost.	5/29-6/7
c. Methods employed for assessing completeness and accuracy of data.	6/7-6/11
11. **Analysis**	
a. Qualitative and quantitative methods used to draw inferences from the data.	6/18-6/26
b. Methods for understanding variation within the data, including the effects of time as a variable.	6/26-6/32
12. **Ethical considerations** - Ethical aspects of implementing and studying the intervention(s) and how they were addressed, including, but not limited to, formal ethics review and potential conflict(s) of interest.	5/14-5/17<br>13/5-13/8
**Results: What did you find?**	
13. **Results**	
a. Initial steps of the intervention(s) and their evolution over time (eg, time-line diagram, flow chart or table), including modifications made to the intervention during the project.	7/2-7/15<br>7/22-7/24
b. Details of the process measures and outcomes.	7/17-7/20
c. Contextual elements that interacted with the intervention(s).	7/26-7/28<br>8/24-8/26<br>9/26-9/33
d. Observed associations between outcomes, interventions and relevant contextual elements.	7/28-10/30
e. Unintended consequences such as unexpected benefits, problems, failures or costs associated with the intervention(s).	10/30-10/34
f. Details about missing data.	11/24-11/27<br>11/33-12/4
**Discussion: What does it mean?**	
14. **Summary**	
a. Key findings, including relevance to the rationale and specific aims.	11/2-11/7
b. Particular strengths of the project.	11/7-11/13
15. **Interpretation**	
a. Nature of the association between the intervention(s) and the outcomes.	11/14-11/17
b. Comparison of results with findings from other publications.	11/14-11/23
c. Impact of the project on people and systems.	11/10-11/13
d. Reasons for any differences between observed and anticipated outcomes, including the influence of context.	12/4-12/7
e. Costs and strategic trade-offs, including opportunity costs.	11/29-11/32

For peer review only

16. **Limitations**	
a. Limits to the generalisability of the work.	11/24-11/27<br>11/33-12/2
b. Factors that might have limited internal validity such as confounding, bias or imprecision in the design, methods, measurement or analysis.	11/27-11/32<br>12/4-12/7
c. Efforts made to minimise and adjust for limitations.	11/7-11/10<br>12/6-12/7
**Conclusions**	
a. Usefulness of the work.	12/10-12/11
b. Sustainability.	12/11-12/15
c. Potential for spread to other contexts.	12/15-12/18
d. Implications for practice and for further study in the field.	12/18-12/21
e. Suggested next steps.	12/21-12/24
**Other information**	
18. **Funding** - Sources of funding that supported this work. Role, if any, of the funding organisation in the design, implementation, interpretation and reporting.	13/1-13/3

Ogrinc G, et al. BMJ Qual Saf 2015;0:1–7. doi:10.1136/bmjqs-2015-004411
Downloaded from http://qualitysafety.bmj.com/ on January 2, 2017

For peer review only

BMJ Open

Assessing the impact of artistic and cultural activities on the health and wellbeing of forcibly displaced people using participatory action research

Journal:	*BMJ Open*
Manuscript ID	bmjopen-2018-025465.R1
Article Type:	Research
Date Submitted by the Author:	01-Oct-2018
Complete List of Authors:	Clini, Clelia; Loughborough University - London, Institute for Media and Creative Industries<br>Thomson, Linda; University College London Division of Biosciences<br>Chatterjee, Helen; University College London, Division of Biosciences
**Primary Subject Heading**:	Qualitative research
Secondary Subject Heading:	Public health, Mental health
Keywords:	creative activities, forced displacement, participatory action research, post-traumatic stress disorder, refugees and asylum seekers

SCHOLARONE™
Manuscripts

Assessing the impact of artistic and cultural activities on the health and wellbeing of forcibly displaced people using participatory action research

Clelia Clini, Linda J Thomson, Helen J Chatterjee

Correspondence to: Helen J Chatterjee, UCL Division of Biosciences, 507B Darwin Building, University College London, London, WC1E 6BT, UK. email h.chatterjee@ucl.ac.uk telephone +44(0)2031084104 fax +44(0)2076797193

Clelia Clini, Institute for Media and Creative Industries, Loughborough University London, UK

Linda J Thomson, UCL Division of Biosciences, University College London, London, UK

Key words: creative activities; forced displacement; participatory action research; post-traumatic stress disorder; refugees and asylum seekers;

Word count: 5080

Abstract

Objective: Drawing upon a growing body of research suggesting that taking part in artistic and cultural activities benefits health and wellbeing, the objective was to develop a participatory action research (PAR) method for assessing the impact of arts interventions on forcibly displaced people, and identify themes concerning perceived benefits of such programmes.

Design: A collaborative study following PAR principles of observation, focus groups and in-depth semi-structured interviews.

Setting. London-based charity working with asylum seekers and refugees.

Participants: An opportunity sample (n=31; 6m) participated in focus groups comprising refugees/asylum seekers (n=12; 2m), volunteers (n=4; 1m) and charity staff (n=15, 3m). A subset of these (n=17; 3m) participated in interviews comprising refugees/asylum seekers (n=7; 1m), volunteers (n=7; 1 m) and charity staff (n=3; 1m).

Results: Qualitative data was analysed using NVivo 11. Focus group findings showed that participants articulated the impact of creative activities around three main themes: skills,

For peer review only - http://bmjopen.bmj.com/site/about/guidelines.xhtml

social engagement, and personal emotions that were explored during interviews. Findings showed that artistic and cultural activities impacted positively on participants by helping them to find a voice, create a support network, and learn practical skills useful in the labour market.

Conclusions: The study expanded on arts and wellbeing research by exploring effects of cultural and creative activities on the psychosocial wellbeing of refugees and asylum seekers. By focusing on the relationship between arts, wellbeing and forced displacement, the study was instrumental in actively trying to change the narrative surrounding refugees and asylum seekers, often depicted in negative terms in the public sphere.

Strengths and limitations of this study:

- Focus on collaborative research
- Different cultural and artistic activities studied
- Researcher spent five months in research setting
- Greater number of female than male participants
- Focus groups attracted relatively low numbers of refugees/asylum seekers

Introduction:

The recent All-Party Parliamentary Group on Arts Health and Wellbeing Inquiry Report shows a growing acknowledgement of the impact of arts and creative practices, stating ‘arts engagement has a beneficial effect upon health and wellbeing and therefore has a vital part to play in the public health arena’.[1 p11] Consensus among practitioners defines wellbeing as ‘the dynamic process that gives people a sense of how their lives are going through the interaction between their circumstances, activities and psychological resources or mental capital’.[2 p3] Rather than depending on a single factor, wellbeing is seen as the result of interplay between different elements, distinguishing personal wellbeing (positive emotion, life satisfaction, vitality, resilience, and self-esteem) from social wellbeing (supportive relationships, trust and belonging).[3] Other authors emphasise ‘resilience and flourishing, rather than just surviving’,[4 p243] a crucial point when investigating the situation of forcibly displaced people who often survive in extreme conditions and whose experience is often discussed in terms of dehumanisation.[5, 6]

Research conducted in the field of forced displacement and mental health shows that ‘asylum seekers and displaced people report high rates of pre-migration trauma’,[6 p275] and

For peer review only - http://bmjopen.bmj.com/site/about/guidelines.xhtml

typically display significant levels of depression, anxiety, post-traumatic stress disorder (PTSD) and non-affective psychoses.[7-11] Refugees and asylum seekers often encounter post-migration living difficulties, such as socio-economic disadvantage, employment barriers, social and emotional isolation, racism and hostility, experience of detention, and uncertainty related to the asylum application process.[7-9, 12] If, as authors suggest,[13-17] there is a strong link between mental health and socioeconomic conditions in terms of employability, income and housing, then the mental health of unemployed asylum seekers living on low incomes in poor quality housing is likely to be worse than that of the general population.

Arts-and-health practitioners believe that ‘aesthetics act upon our senses to make us feel more, hear more and see more than we otherwise might… feelings are intertwined with mental, physical, spiritual and social health’.[18 p4] Several studies explored the relationship between arts, health and wellbeing and a growing body of evidence suggests that cultural participation (including music, art making, theatre, dance, museum and heritage activities) enhances human health and wellbeing.[1, 19-27] Research conducted in museums-and-health, for example, suggests that ‘museum and art gallery encounters can help with a range of health issues, enhance wellbeing, and build social capital and resilience’.[27 p286] According to research conducted in Norway with over 50,000 adults,[28] participation in both receptive/passive and creative/active cultural activities was significantly associated with good health, life satisfaction, and low anxiety and depression. Findings demonstrated how manual creative practices such as knitting had a positive impact on people suffering from depression and post-traumatic stress disorder because ‘the movements involved in knitting are bilateral, rhythmic, repetitive, and automatic’.[28 p40] The authors attributed the positive mood of knitters to enhanced production of serotonin resulting from repeated movements, and that bilateral processes appeared to engage brain capacity and facilitate a meditative-like state more readily than unilateral ones. Although creative activities are seen to provide major benefits for refugees and asylum seekers, researchers have become ‘increasingly conscious of the value of recording and analysing what has been happening’.[8 p1]

The current study was conducted in the light of mental health and arts-in-health/museums-in-health evidence, and the large number of organisations that engage migrants, refugees and asylum seekers in the arts, with nearly 200 of these in the UK.[29] The current research was conducted at the Helen Bamber Foundation (HBF), a charity offering support to refugees and asylum seekers as victims of torture and human rights violations. HBF adopts a holistic approach to support its clients that includes cultural activities within a three-phase model of integrated care comprising stabilisation (medical and legal support), intervention (trauma-

For peer review only - http://bmjopen.bmj.com/site/about/guidelines.xhtml

focussed therapy and general psychological care), and integration (attending the creative, computing and English classes, and interacting with other clients). The model is based on the needs of refugee/asylum seekers who often require assistance on several fronts (e.g. psychological support might not be effective if a person does not have a place to sleep or lives in a dangerous situation, though giving people a place to sleep is not enough to overcome trauma). Their creative arts programme (CAP) led by volunteers includes art (painting and drawing), photography, textiles (dressmaking and knitting) and singing, and is attended by c.100 clients. The study took the view that arts participation would enhance the sense of wellbeing by allowing participants to form meaningful relationships based on trust and mutual support (improving social wellbeing) and enhance self-esteem and self-confidence (increasing personal wellbeing).

To provide voice and agency for participants, the study used a collaborative approach to explore client experiences of creative activities with HBF clients, staff and volunteers acting as co-researchers. The research was operationalised according to the principles of participatory action research (PAR)[30, 31] as a ‘way of opening up space for dialogue and conversation’,[32 p28] aiming to ‘understand and also improve a particular situation’,[32 p1] both pertinent aspects when working with displaced people. The PAR approach encourages an active contribution in the production of knowledge within a collaborative framework, emphasising ‘equal partnerships’,[33 p167] and the ‘role of the participant in the design, implementation, and dissemination of the research’.[34 p78] The collaborative character of PAR is grounded in efforts to ‘democratise the research process’,[35 p3] so ‘equal weight and consideration is given to the contributions of both the community and academic partners’[36 p170] again valuable for everyone involved but specifically refugees/asylum seekers. One of the pillars of PAR, especially when community-based is ownership.[37] By offering equal weight to client contributions focusing on the relationship between forced displacement and arts participation, the study aimed to empower participants by developing their sense of ownership in the project. Through collaborative working between the charity, displaced people and academics, the purpose of the research was to co-develop a methodological approach to address the needs of refugees/asylum seekers and to co-produce interview questions that could be applied in UK and international settings. The objectives were to expand on arts-in-health and collaborative research approaches by exploring the benefits of engagement in cultural and creative activities on the health and wellbeing of forcibly displaced people and contribute to the current debate on migration and public health.

For peer review only - http://bmjopen.bmj.com/site/about/guidelines.xhtml

Method

Design

Qualitative research conducted comprised observation, focus groups and one-to-one semi-structured interviews with clients, volunteers and staff as co-researchers within a collaborative PAR approach.[38]

Participants

The study recruited an opportunity sample of volunteer participants through information leaflets and contact details in reception. Clients were approached by the researcher using two recruitment criteria: i) clients had received therapy for at least two years prior to the research and had entered the integration stage of the model of integrated care; this meant that though still vulnerable, clients had established a therapeutic relationship of trust enabling them to form safe relationships in the wider community and move forward with their lives; and ii) they had attended at least one of four CAP groups (art, photography, singing and textiles) for around two years. All participants spoke English sufficiently well to participate having attended English classes for about two years as part of integrated care. In total, 31(6m) participants volunteered for the study and attended focus group comprising 12(2m) refugees/asylum seekers, 4(1m) volunteers, and 15(3m) charity staff. Of these, 17(3m) participated in interviews comprising 7(1m) refugees/asylum seekers, 7(1m) volunteers, and 3(1m) staff.

Patient and public involvement

HBF staff were instrumental in the application for research funding and collaborated with the researcher to determine the study's objectives. HBF clients were involved in the recruitment process by recommending that other clients join them in the research, though the researcher ensured that they matched recruitment criteria (above). In keeping with PAR, research questions and outcome measures concerning the effects of cultural and creative activities on the psychosocial wellbeing of refugees/asylum seekers were determined by co-researchers (HFB clients, volunteers and staff) with differing involvement depending on their role at the charity. Focus groups and interviews gave voice to clients' priorities and preferences but, due to their vulnerability, no personal information was requested as the process of remembering could have been difficult.[7, 10] Although some participants mentioned personal issues and experiences, they were not encouraged to do so. Data generated by co-researcher involvement

For peer review only - http://bmjopen.bmj.com/site/about/guidelines.xhtml

in the research collaboration informed focus groups, in turn focus group outcomes led to the development of questions for the in-depth interviews to follow. In addition to participation in focus groups and interviews, a core group of seven co-researchers (3 clients, 2 volunteers, 2 staff, with 1 male per category) were involved in design of the interview guides, discussion of emerging themes and dissemination of the research findings to other participants for verification and comments, in accordance with PAR principles.[33] The results of the research were fed back to everyone involved with HBF through on-going communication with the researcher, an internal report lodged at the charity, and an end-of-project event.

Data collection

Data collection carried out by the location-based researcher consisted of three stages: Stage 1 (months 1-2) participant observation; Stage 2 (months 3-4) focus groups; and Stage 3 (month 5) semi-structured interviews. In Stage 1, participant observation was used as a starting point 'for studying how organisations work, the roles played by different staff and the interaction between staff and clients'.[38 p32]. The researcher attended creative classes for clients and spent a day a week working from HBF (12-15 hours per week) to determine the nature of focus groups. In Stage 2, four focus groups were held, first and final with clients (n=8, n=4 respectively), second with volunteers (n=4), and third with staff (n=15). In the focus groups, participants were asked to discuss research questions (Table 1) involving ways of gathering information on the impact of creative activities, and reasons for attending groups (or in the case of volunteers and staff, reasons for involvement). All focus groups were organised informally to facilitate development of discussion among participants and allow an exchange of experiences and ideas. Ideas that emerged during focus groups laid the basis for first drafts of interview questions, tailored to clients, volunteers or staff (Table 2). In line with the collaborative ethos, drafts were circulated to receive feedback from co-researchers prior to the interview stage, and volunteers and staff were consulted and asked to provide comments and suggestions for interview questions. For Stage 3, clients (n=7), volunteers (n=7) and staff (n=3) participated in interviews with the researcher using a semi-structured format to allow free expression within the research constraints and limit potential research bias.[39, 40]

INSERT TABLE 1 ABOUT HERE

INSERT TABLE 2 ABOUT HERE

For peer review only - http://bmjopen.bmj.com/site/about/guidelines.xhtml

Data analysis

A first informal thematic analysis was conducted by the researcher while writing field notes consisting of a chronicle of descriptive rather than analytic events both observed and provided by co-researchers as the 'raw material of the research'.[30 p38] The process of transcribing notes was used to identify key points connected to the research question. This preliminary analysis informed focus group topics where points were discussed more in detail. After the first round of focus groups, all notes were compiled into a Word document and repeatedly read by the researcher and co-researchers to search for recurring topics and themes. Thematic analysis of focus group outcomes were explored in detail during interviews. All interviews were recorded and transcribed to become familiar with the data and begin the coding process [37]. All details in the transcripts were recorded verbatim (e.g. sighs, laughter, silences, and tears) as their exclusion could have changed the meaning expressed.[30] Transcriptions were uploaded into NVivo11 to produce nodes from coding-relevant concepts. NVivo's word frequency query facilitated the search for key words (including stem words and synonyms) grouped under the same theme. The text search query provided a comprehensive analysis of data. After coding of data into initial themes,[37] a review of themes followed to refine the analysis.

Results

Focus Groups

Focus group data were analysed using thematic analysis (NVivo11). Findings showed consistency in the way all HBF participants (clients, volunteers and staff) articulated their reflections on the impact of creative activities. The benefits of creative activities were highlighted in clusters emerging from around three main overarching themes: 'social aspects/friendships', 'skills', and 'mood-personal sphere'; the latter sub-divided into 'brain' (creative thought processes), 'routine', self-expression' and 'confidence'. During focus groups, co-researchers were asked to write answers to the questions posed, and a collective discussion followed. Clients responded consistently about benefits of the creative programmes. One client listed 'Activities help my brain think about other stuff and keep me busy and relax. You can't think about bad things. To learn more skills. I can use skills to help others.' Another noted 'Give me confidence. Make me feel good and useful. Change my mood. Learn new things and meet people. Improve my skills. Give me hope to lead a better life'. Similarly, a client wrote 'Allows me to feel included. Safe environment. Improves my mood. Learn new skills. Gets me out of the house. Meet new people'. Responses illustrate

For peer review only - http://bmjopen.bmj.com/site/about/guidelines.xhtml

that clients perceived their involvement as beneficial at multiple and overlapping levels. Enhancement of mood appeared to derive from not only meeting new people, forging meaningful relationships and feeling part of a group but also was connected to a growing self-confidence and self-esteem experienced as clients acquired new skills.

The focus group with HFB staff emphasised that being busy in the company of others allowed clients to feel safe and not to dwell upon their own situation. As one staff member observed 'Creative activities allow clients to momentarily leave out their problems or memories; by focusing on a particular activity, they cannot think about anything else'. Staff recognised the social aspect of activities as a major benefit, with one suggesting 'in these groups, clients learn to have balanced, reciprocal relationships, which they have not experienced before or for a long time given their experience of violence (trafficking). They provide clients with an alternative identity, i.e. they are not simply victims here, they are learners, artists, dressmakers, etc.' Similar themes emerged during focus group with volunteers. A female volunteer at HBF for two years suggested that activities were beneficial because 'Participants do not feel assessed as clients, they are just learners or artists. These groups pave the way for identifications different from being a victim, a refugee, or an asylum seeker'. A relatively new male volunteer, involved in the organisation four months, was impressed by the solidarity between clients and emphasised as beneficial the 'feeling of belonging to a group' and 'strong support that people receive from other members'.

Interviews

During interviews, clients elaborated on practical and social skills, and commented on the positive effect of attendance on their mood and emotion:

Practical skills: Learning new practical skills or improving those previously learnt emerged strongly among asylum seekers without the right to work. As a client pointed out:

I have learned a lot of skills: I've learned sewing, now I'm learning art, I can do better, before I didn't know how to draw a person, to mix colours, you know such things... so you keep on learning, slowly by slowly, then in the end you find yourself, you're a professional, so that is very great... to me... I like to come, always and attend, and listen to what they tell me, and I do it.

Or, as another client explained:

I've learned new skills [...] and you can never waste your time learning new skills. New skills always help you in your life, always. So, everything I've learned will help me. You

For peer review only - http://bmjopen.bmj.com/site/about/guidelines.xhtml

think like 'film club, learning how to edit, etc.'. People pay people on YouTube to edit their films, and I'm, like, I could just do my own, if I wanted to have a YouTube channel, I can do my own.

Skill improvement provided a sense of achievement and improved self-confidence, talking about learning new skills, one client observed:

When you are in this situation it feels like life has, in a way, stopped. And you can't do anything to change it. But by doing all this activity you feel, or I felt…it was like, you know, that I was learning something in my life, rather than just waiting.[…] For instance, if I go for a job somewhere, where you have to write what skills you have, so I could include all of this. I mean I don't have a certificate or, like, proper qualifications, but I've learned all those skills, so I feel like, it is, in a way, impressive? Because you feel like, you know, rather than just waiting and not doing anything, you have been learning.

The sense of self-improvement associated with learning skills was linked to the perception that by learning, clients were preparing themselves for a new life when they would be allowed to live and work in the UK, countering the perception that life for them had stopped and giving them something to look forward to. The process of skills learning was not simply a vertical one; peer learning was equally important, stressing the importance of social aspects.

Social skills: The opportunity to overcome social isolation and create a social network emerged as one of the benefits of creative engagement. As one client indicated:

The groups changed me because you know when I come, I feel lonely and doesn't have anybody. I was lonely I don't even know whether there will be a home from morning to live in. But when I started coming these groups, I told you I feel active, I feel coming to see people, that I speak to. I don't even know how to communicate with people before. I told you because of my coming here I now feel to communicate to people. Before I never communicate to people, I don't know how to do it. And I don't know how to play with people. But these groups make me now taught me: play with people, to meet me.

The client expressed the difficulties of establishing new relationships and the feeling that the group was a place in which they could regain this ability to connect with other people. The social aspect was an important point reiterated in all interviews; fellow clients were described as 'friends' but also as 'brothers and sisters'. Arts activities allowed clients to create a community characterised by solidarity, as one client explained:

For peer review only - http://bmjopen.bmj.com/site/about/guidelines.xhtml

If you're in a situation where you've been completely isolated from people for a while and you just don't know who to trust, or to be around people, it's one of those spaces where you can get to meet people, socialise, and actually make friends.[…] And these friendships do last. A life-time, because you understand what the person has gone through, you don't have to explain a lot, you know, they just know that you've been through something terrible.[…] So, it's easier when you have people here who just understand what you've possibly been through.

This remark connects with the observation that 'engagement in participative creative arts activities in communities can help to build social capital, address loneliness and social isolation, and build personal confidence and a sense of empowerment'.[25 p32] The awareness of experiencing similar situations makes clients feel free to share their own experiences with one another but also not to, if they did not feel like opening up:

There is a sense of community: you know you go there, you know there are people like you in the same situation you're not going to be judged, so it's that sense of community: we know what's going on, we don't have to talk about it.[...] It's a distraction from immigration and we know we are all going through it, but we don't have to talk about it. There are other things going on in life, and we talked about, for example in the art group, the works we produced and what we could do, what we could achieve and get inspired by each other's work.

Again, this remark introduced the third broad theme which emerged out of the interview analysis, which was the impact of creative arts on personal mood and emotion.

Mood and Emotion: Learning or improving skills while forging long-lasting relationships in a context of deep social isolation (outside of HBF), inevitably had a substantial impact on mood and emotion. In discussing this aspect of creative arts, interviewees focused particularly on how groups allowed them to have a routine, a 'luxury' one client said 'when you can't do nothing but waiting for the government to decide on your right to stay in the country'. Routine combined with skills learning/improving was thought to have a positive impact on clients' mental health; 'having something to do' and 'something to look forward to' were recurrent expressions during interviews. As one client noted:

I used to come here every day of my life: Monday, Tuesday, Wednesday... because I don't have anywhere to go, so I just come here, and I attached myself to the groups, we met, we talk, we... then I've gone to knit, I've gone to do computer, reading, many things I learnt here, so I just like it.

For peer review only - http://bmjopen.bmj.com/site/about/guidelines.xhtml

Another client elaborated further:

> *It's something that I look forward to. One thing you have to bear in mind is that I am not working, and I am not studying, you know, so it was the only thing that I would look forward to because it was something to do, otherwise I would just be at home, doing nothing, you know, feeling very sorry for myself, getting upset all the time. I mean I still feel that way but at least there's something to look forward to, when you don't have hope or something to look forward to, it increases your depression levels, so for me it's helped me greatly.*

This statement describes the social and emotional isolation as part of post-migration living difficulties. The routine of classes, together with an awareness that others in similar situations would attend, provided comfort to clients, countering feelings of isolation:

> *There is nothing else for you and it's also a way of, like, using your time wisely, 'cause at the end of the day most of the time you're just sitting down doing nothing, and doing nothing slowly begins to affect your mind, your brain, you know you become lazy, you know you literally are just in a four-squared room[…]*

The benefits of a routine were increased by inclusion of creative activities that according to clients and volunteers, had a positive impact on mood because they exercised the brain and allowed clients to find a new language to express themselves. As a client stated:

> *[Attending creative groups] gives you a lot of confidence, you know. And it allows your brain to think outside of the box because I think we are just in that box and... but when you attend these classes, your mind, you know, opened and you learn new things and you want to then learn other things as well.*

This comment highlights an important link between arts and feelings; as another stated:

> *It is more of expressing, more of letting go, it's like getting a spirit out of you, and you don't have necessarily have to tell someone 'I've done this because of that and that, and this', you know. Because sometimes you just don't find the voice to talk about it and, I, as a person am really shy and, you know, I feel easily embarrassed, you know.[...] So... that's why I'm into arts, yes I'm doing arts really.*

Clients agreed that the possibility of self-expressing without having to articulate their feelings, helped them grow emotionally and gain confidence. It was not only clients who benefited from CAP; findings also pointed to the positive impact on volunteers, a point stressed by all volunteers when discussing personal experiences.

For peer review only - http://bmjopen.bmj.com/site/about/guidelines.xhtml

Discussion

Creative and cultural activities were observed over a sustained period of five months. Clients participating in the activities had reached the integration stage of the model of integrated care consequently had been taking part in CAP activities for two plus years prior to the research. Activities provided refugees and asylum seekers with new skills, including practical and technical skills, and social and life skills involving language acquisition obtained partly through informal peer learning and mutual support. Learning new skills contributed to the sense of wellbeing and empowerment experienced by HBF clients. The time spent by the researcher at the HBF was vital for familiarisation with the context, getting to know people involved in the organisation, and explaining the research to recruit participants from among clients, volunteers and staff. The fact that several clients attended more than one group per week (2-4) made it possible to forge trusting relationships and recruit co-researchers, allowing them to feel comfortable in speaking their mind. A possible issue here was that the number of groups attended by clients was not accounted for, so it was not evident as to whether increased participation furthered benefits at a higher/faster level. Once recruited, participants remained with the study in their co-researcher roles and continued to attend activities on a weekly basis for the five months (except for rare absences due to illness).

Activities appeared to positively enhance mood and emotion both for clients and volunteers facilitating CAP groups. Although passive participation was not compared,[28] active and creative participation in the groups specifically benefited the clients. A key reason was that asylum seeker status in the UK would not have permitted alternative occupation or employment, so clients might not have otherwise left their homes or met people on a regular basis. In keeping with previous research, creative and cultural participation led to improved social health,[18] and aligned with a growing body of evidence[1] to suggest that creative activities enhanced mental wellbeing,[19-27] helping clients to develop self-confidence and resilience.[21] Client preferences for activities indicated that singing was their favourite group, followed by photography, art and textiles. Singing was regarded as beneficial because it allowed people to meet socially and work collaboratively, aspects considered important in countering loneliness. Findings aligned with a recent qualitative study indicating that choral singing promoted improvements in social, emotional, physical, and cognitive functioning and that benefits were experienced similarly irrespective of age, gender, nationality.[41] In the current study, photography, art and textiles were praised by clients for the skills taught and although social aspects were relevant, skills that allowed participants to continue activities at home (e.g. sewing, drawing) were highly valued. Interviewed staff were asked to comment

For peer review only - http://bmjopen.bmj.com/site/about/guidelines.xhtml

on the popularity of activities, and although the order was the same as that of clients, they were not asked their opinion on which were most beneficial.

The current study bridged the gap between two research disciplines: arts-in-health and forced displacement and mental health. It contributed to existing literature by demonstrating beneficial effects of creative activities on the wellbeing of refugees/asylum seekers and that PAR was an appropriate method to use for collaboration between displaced communities and academics. The PAR approach was particularly useful when conducting research with refugees/asylum seekers suffering from PTSD and other mental health issues because the process of involvement as co-researchers helped to counter client isolation and build social capital through regular meetings. In helping to interpret findings, clients acquired research methods skills (e.g. drafting interview guides, conducting focus groups, discussing data) and organisational skills (event/exhibition). Participating in the research and disseminating findings gave clients a sense of agency and ownership of the project that enhanced self-confidence and feelings of empowerment and provided a positive means of expression countering their refugee/asylum seeker status that effectively placed them powerless.

In terms of study weaknesses, the greater number of female than male participants was probably due to the fact that most creative groups were led by female volunteers (2m, 8f). Most clients attending these groups were also female except for singing, where numbers were approximately equal (although only one male choir member took part in a focus group). Given the nature of the singing activity, conversation was limited to the first minutes before arrival; once rehearsals had started it was difficult for the researcher to form relationships with clients. It was also possible that some activities were seen by clients as gender-neutral (e.g. photography or singing), but others such as working with fabrics and soft materials (e.g. wool and cloth) may have been seen as a predominantly female activity; male participants might have been more comfortable working with harder materials (e.g. wood and metal). It was generally more difficult to engage male participants in the research; a possible reason for this was female clients might have found it easier to relate to the female researcher. A further weakness was low attendance for focus groups possibly due to difficulties of organising them at convenient times for everyone, given changing client circumstances (e.g. interviews for benefits, housing etc.). More clients expressed an interest than those who took part in the study, nevertheless were happy to informally discuss the topics debated. Others wanted to be kept in the loop about the research without participating in focus groups and interviews, so methods and these were discussed informally during meetings between researcher and clients.

For peer review only - http://bmjopen.bmj.com/site/about/guidelines.xhtml

Conclusions

This study expanded on arts-in-health and collaborative research approaches by exploring the effects of cultural and creative activities on the health and wellbeing of refugees and asylum seekers. Creative activities at HBF provided opportunities for clients to socialise and develop new relationships based on respect and mutual recognition. In turn, these opportunities countered loneliness and allowed refugees/asylum seekers to develop a sense of belonging in a safe space. The opportunities further supported freedom of expression and the possibility of developing a new identity to counter the negative stereotype of being viewed as a refugee or asylum seeker,[42] leading to sustained improvements in positive mood and self-confidence, and countering anxiety, depression and other mental health issues. By focusing on the relationship between arts, wellbeing and forced displacement and in keeping with PAR, the study actively changed the narrative surrounding refugees/asylum seekers and gave an understanding of how to improve conditions for forcibly displaced people through opportunities for social connectivity, creative thinking and employment skills. It is recommended that other refugee/asylum seeker organisations could utilise the interview questions generated by the study, or develop their own following PAR guidelines, to appraise and improve their migrant provision particularly in terms of up-skilling peer-learning. If scaled up across the UK, programmes of creative activities for migrants could reduce pressure on health and social care services and help to address the social determinants of health inequality.[16] Research outputs have wider implications for policy development, for example by the United Nations Refugee Agency, World Health Organisation, and other government and non-government agencies.

Contributors

CC, LT and HC made substantial contributions to the conception and design of the work; drafting and critically revising the work for intellectual content; and the final approval of the version published. CC conducted the acquisition, analysis and interpretation of data for the work. All authors agree to be accountable for all aspects of the work. We would like to thank the clients, volunteers and staff who were co-researchers in the project.

Funding

The work was supported by the Global Challenges Research Fund (GCRF) Grant reference: ES/P003818/1 PI: HC

For peer review only - http://bmjopen.bmj.com/site/about/guidelines.xhtml

Ethics

The study gained approval from UCL Ethics Committee (Ethics Application 4526/002 Co-developing a method for assessing the psychosocial impact of cultural interventions with displaced people: Towards an integrated care framework) to carry out the research with potentially vulnerable participants.

Competing interests

The authors have no competing interests.

Data sharing statement

Due to the confidential nature of the human participant data, it will not be freely available via free access databases. Any request for data should be addressed to the corresponding author.

References

1. All-Party Parliamentary Group on Arts, Health and Wellbeing (APPGAHW). Creative Health: The Arts for Health and Wellbeing. Inquiry Report. 2017; http://www.artshealthandwellbeing.org.uk/appg-inquiry/
2. New Economics Foundation (NEF). 2009. National accounts of wellbeing: What is wellbeing? NEF. http://www.nationalaccountsofwellbeing.org/learn/what-is-well-being.html
3. New Economic Foundation (NEF). National Accounts of Well-being: Bringing Real Wealth onto the Balance Sheet. 2009. https://neweconomics.org/uploads/files/2027fb05fed1554aea_uim6vd4c5.pdf
4. Ander E, Thomson LJ, Noble G, Lanceley A, Menon U, Chatterjee H. Generic well-being outcomes: towards a conceptual framework for well-being outcomes in museums. Museum Management Curatorship 2011; 26:237–259. doi:10.1080/09647775.2011.585798
5. Crawley H, Düvell F, Jones K, MCMahon s, Sigona, N. Destination Europe? Understanding the dynamics and drivers of Mediterranean migration in 2015. MEDMIG Final Report 2016. www.medmig.info/research-brief-destination-europe.pdf
6. Vaughan-Williams N. “We are *not* animals!” Humanitarian border security and zoopolitical spaces in Europe. Political Geography 2015;45:1–10. doi:10.1016/j.polgeo.2014.09.009

For peer review only - http://bmjopen.bmj.com/site/about/guidelines.xhtml

7. Katona C. Non-affective psychosis in refugees. Br Med J 2016;352:i1279. doi:10.1136/bmj.i1279
8. Robjant K, Robbins I, Senior V. Psychological distress amongst immigration detainees: A cross-sectional questionnaire study. Brit J Clin Psych 2009;48:275–286. doi:10.1348/014466508X397007
9. Alpak G, Unal A, Bulbul F, Sagaltici E, Bez Y, Altindag A et al. Post-traumatic stress disorder among Syrian refugees in Turkey: A cross-sectional study. Int J Psych Clinic Practice 2015;19:45–50. doi:10.3109/13651501.2014.961930
10. Schubert CC, Punamäki R. Mental health among torture survivors: cultural background, refugee status and gender. Nordic J Psych 2011;65:175–182. doi:10.3109/08039488.2010.514943
11. Silove D, Sinnerbrink I, Field A, Manicavasagar V, Steel Z. Anxiety, depression and PTSD in asylum-seekers: associations with pre-migration trauma and post-migration stressors. Brit J Psych 1997;170:351–357. doi:10.1192/bjp.170.4.351 doi:10.1348/014466508X397007
12. Aragona M, Pucci D, Mazzetti M, Geraci S. Post-migration living difficulties as a significant risk factor for PTSD in immigrants: a primary care study. Italian J Public Health 2012;9:e7525;1–8. doi:10.2427/7525
13. Allen J, Allen M. The social determinants of health, empowerment and participation. In S Clift, P Camic (Eds.), Oxford Textbook of Creative Arts, Health, and Wellbeing: International Perspectives on Practice, Policy and Research. New York, Oxford: Oxford University Press; 2016, p27–34.
14. Fisher M. Why mental health is a political issue. The Guardian, July 16, 2012. https://www.theguardian.com/commentisfree/2012/jul/16/mental-health-political-issue
15. Griffin J. The Lonely Society. London: The Mental Health Foundation. https://www.mentalhealth.org.uk/sites/default/files/the_lonely_society_report.pdf
16. Marmot M, Allen J, Goldblatt P, Boyce T, McNeish D, Grady M et al. Fair Society, Healthy Lives: The Marmot Review. 2010; http://www.instituteofhealthequity.org/resources-reports/fair-society-healthy-lives-the-marmot-review/fair-society-healthy-lives-full-report-pdf
17. Paul K, Moser K. Unemployment impairs mental health: Meta-analyses. J Vocational Behavior 2009;74:264-282. doi:10.1016/j.jvb.2009.01.001
18. Prior RW. Editorial. J Applied Arts Health 2009;1:3–6.

For peer review only - http://bmjopen.bmj.com/site/about/guidelines.xhtml

19. Bygren LO, Johansson S, Konlaan BB, Grjibovski AM, Wilkinson AV, Sjöström M. Attending cultural events and cancer mortality: A Swedish cohort study. Arts Health: Int J Res Pol Pract 2009;1:64–73. doi:10.1080/17533010802528058
20. Camic P, Chatterjee H. Museums and art galleries as partners for public health interventions. Perspect Public Health 2013;133:66–77.
21. Chatterjee H. Museums and art galleries as settings for public health interventions. In S Clift, P Camic (Eds.), Oxford Textbook of Creative Arts, Health, and Wellbeing: International Perspectives on Practice, Policy and Research. New York, Oxford: Oxford University Press 2016; p.281–289.
22. Clift S, Skingley A, Coulton S, Rodriguez J. The effectiveness and cost-effectiveness of a participative community singing programme as a health promotion initiative for older people: protocol for a randomised controlled trial. BMC Public Health 2011;11:1–6. doi:10.1186/1471-2458-11-142
23. Clift S, Camic P. Oxford Textbook of Creative Arts, Health, and Wellbeing: International Perspectives on Practice, Policy and Research. New York, Oxford: Oxford University Press, 2016.
24. Konlaan BB, Bygren LO, Johansson S. Visiting the cinema, concerts, museums or art exhibitions as determinant of survival: a Swedish fourteen-year cohort follow-up. Scand J Public Health 2000;28:174–178. doi:10.1177/14034948000280030501
25. Staricoff RL. Arts in health: A review of the medical literature. London: Arts Council England, Research Report 2004;36:1–91. http://www.artsandhealth.ie/wp-content/uploads/2011/08/AHReview-of-Medical-Literature1.pdf
26. Staricoff RL. Arts in health: The value of evaluation. Perspect Public Health 2006;126:116–120. http://journals.sagepub.com/doi/pdf/10.1177/1466424006064300
27. Chatterjee HJ, Noble G. Museums, Health and Wellbeing. Farnham: Ashgate, 2013.
28. Cuypers K, Krokstad S, Holmen TL, Knudtsen MS, Bygren LO, Holmen J. Patterns of receptive and creative cultural activities and their association with perceived health, anxiety, depression and satisfaction with life among adults: the HUNT study, Norway. J Epidemiol Community Health 2012;66:698–703. https://jech.bmj.com/content/jech/66/8/698.full.pdf
29. Kidd B, Zahir S, Khan S. Arts and Refugees: History, Impact and Future. London: Arts Council England, 2008. https://baringfoundation.org.uk/wp-content/uploads/2014/10/ArtsandRefugees.pdf

For peer review only - http://bmjopen.bmj.com/site/about/guidelines.xhtml

30. Kemmis S, McTaggart R, Nixon R. The Action Research Planner: Doing Critical Participatory Action Research. Singapore: Springer 2014.
31. Bradbury H. Introduction: How to Situate and Define Action Research. In H Bradbury (Ed). The Sagw Handbook of Action Research, London: Sage 2015. p.1–9.
32. Nicolaidis C, Raymaker DM. Community based participatory research with communities defined by race, ethnicity, and disability: Translating theory to practice. In H Bradbury (Ed.), The Sage Handbook of Action Research. London: Sage 2015. p.167–178.
33. Daykin N, Stickley T. The role of qualitative research in arts and health. In S Clift, P Camic (Eds.), Oxford Textbook of Creative Arts, Health, and Wellbeing: International Perspectives on Practice, Policy and Research. New York, Oxford: Oxford University Press 2016, p.73–82.
34. Vaughn LM, Jacquez F. Community-Based Participatory Research Studies Involving Immigrants. In S Coughlin, SA Smith, ME Fernandez (Eds.), Handbook of Community-Based Participatory Research. New York, Oxford: Oxford University Press, 2017. p.115–130.
35. Blumenthal DS. Is Community-based participatory research possible? Am J Prev Med. 2011;40:386–389.
36. Braun V, Clarke V. Using thematic analysis in psychology. Qual Res Psychol 2006;3:77–101.
37. Pope C, Mays N. Qualitative Research in Health Care, 3rd Edition. Oxford: Blackwell, 2006.
38. Pope C, Ziebland S, Mays N. Analysing Qualitative Data. In C Pope, N Mays. Qualitative Research in Health Care, 3rd Edition. Oxford: Blackwell. 2006. p.63–81.
39. Kemmis S, McTaggart R, Nixon R. The Action Research Planner: Doing Critical Participatory Action Research. Singapore: Springer 2014.
40. Bradbury H. Introduction: How to Situate and Define Action Research. In H Bradbury (Ed). The Sage Handbook of Action Research, London: Sage 2015. p.1–9.
41. Livesey L. Morrison I. Clift S. Camic P. Benefits of choral singing for social and mental wellbeing: Qualitative findings from a cross-national survey of choir members. J Pub Mental Health 2012;11:10–26.
42. Khosravi S. 'Illegal' Traveller: An Auto-ethnography of Borders. Basingstoke: Palgrave MacMillan 2010.

For peer review only

Table 1. Focus group questions

Clients:
1. Why do you like the HBF creative arts programme?
2. How would you gather information or evidence about the benefits of artistic activities?
3. Which creative arts classes have a positive impact on the lives of refugees?
Circle the option(s) that you think work best or add another one.
Arts and crafts   Drawing   Films   Knitting   Photography   Singing   Textiles.
Volunteers:
1. Why do you volunteer at the HBF?
2. How do you think your group benefits clients?
3. How would you collect evidence on the effects of participating in artistic activities?
Staff:
1. Why do you recommend clients to attend Creative Arts groups?
2. What do you think are the benefits of attending arts groups?
3. How would you collect evidence on the effects of participating in artistic activities?

For peer review only

Table 2. Interview questions

Clients:

1. How long have you been attending the CAP classes for?
2. Which groups do you attend?
3. Why did you choose this/these groups(s)?
4. What do you like the most about these groups?
5. Is there anything about the groups that you find difficult?
6. Anything you would like to change?
7. Is there a group you prefer? Which one? Why?
8. How do you feel when you attend this group?
9. Do you think there are benefits of attending these groups? If so what are they?
10. During our previous meeting, it emerged that participants feel that groups allow them to learn new or improve already acquired skills. Do you agree? Have you learnt any new skill? And what do you think about the possibility of learning new skills? (How does that impact your life)
11. Another element which emerged during our meeting is that people enjoy the social aspect of these groups: meeting people and finding new friends. What do you think about this? [supporting questions: do you like being around other people? Why? What happens when you attend a session? Have you made new friends? How do you feel about that?]
12. Among all of the reasons why you enjoy attending the creative arts group, which one is the one that you feel strongest about?
13. Why do you keep attending?
14. If you could compare the way you were feeling before joining any of these activities and the way you feel now, would you say you feel any different? Explain
15. Creative activities of course are part of the support that HB offers to its clients. Do you think that you would feel the same about yourself today even without attending these creative activities groups? Why?
16. Do you think that taking part in these creative activities has had any influence on the way you see yourself? And the way you see yourself in London?
17. If you were to recommend someone to join a group, what would you say?

For peer review only - http://bmjopen.bmj.com/site/about/guidelines.xhtml

Volunteers:

1. What do you teach?
2. How long have you been volunteering for?
3. Why did you decide to volunteer?
4. How did you learn about the Helen Bamber Foundation?
5. What do you think your group offers clients?
6. What are the main challenges you face/have faced as a volunteer?
7. Do you find it difficult to engage clients in the activity you coordinate? What do you think clients like about the way you (and your colleagues) handle the sessions? Is there anything that clients don't like?
8. How would you describe a typical session?
9. What do you think people like the most about your group?
10. Do you ever discuss emotions with clients? Or their personal situation?
11. Look at the notion of wellbeing defined by NEF:

Wellbeing as 'the dynamic process that gives people a sense of how their lives are going through the interaction between their circumstances, activities and psychological resources or 'mental capital.' (NEF 2008 p3).

According to the NEF, there are different components to wellbeing, such as:
'Personal wellbeing (emotional wellbeing, satisfying life, vitality, resilience, self-esteem and positive functioning) and social wellbeing, including supporting relationships and trust and belonging.' (NEF 2009).

12. Do you think that the activity you lead has any impact on the wellbeing of clients? Elaborate
13. If you were asked to recruit people for your group, what would you say to convince them to join?
14. Would you use different words to promote your group with men and women? Why?
15. Do you think that your group makes a difference in the lives of clients? How? Why?
16. And what about your own life? What is the impact of your volunteer activity on your own life?

For peer review only - http://bmjopen.bmj.com/site/about/guidelines.xhtml

Staff:

1. How long have you been working at HB for?
2. Why did you decide to work here?
3. What is your role?
4. As part you work, do you have any direct contact with clients?
5. Could you say a few words on your relationship with clients?
6. How would you describe your own experience at HB so far?
7. What are the main challenges that you face/have faced in your position?
8. Do you think that your own gender influences the ways in which clients relate to you?
9. In your position, do you discuss clients' personal situations and feelings?
10. If so, do you think that the cultural background of clients affects the way in which you discuss emotions? If it does, how and why?
11. What are the main challenges of discussing emotions with clients?
12. Could you say a few words on the Model of Integrated Care? Do you think it works? And if so, why?
13. Do you recommend clients to attend any of the CAP groups? What do you say in this case?
14. What role do you think cultural and creative activities play in the recovery of clients?
15. Are there any particular activities that, in your opinion, are more popular with clients?
16. Would you say that the programme makes a difference in the lives of clients? If yes, please elaborate
17. Have you witnessed clients benefiting from their own participation in the creative arts programmes? If yes, in what ways?

For peer review only

Research and reporting methodology

Revised **Standards for Quality Improvement Reporting Excellence** (**SQUIRE 2.0**) publication guidelines

Notes to authors

- The SQUIRE guidelines provide a framework for reporting new knowledge about how to improve healthcare.
- The SQUIRE guidelines are intended for reports that describe system level work to improve the quality, safety and value of healthcare, and used methods to establish that observed outcomes were due to the intervention(s).
- A range of approaches exists for improving healthcare. SQUIRE may be adapted for reporting any of these.
- Authors should consider every SQUIRE item, but it may be inappropriate or unnecessary to include every SQUIRE element in a particular manuscript.
- The SQUIRE glossary contains definitions of many of the key words in SQUIRE.
- The explanation and elaboration document provides specific examples of well-written SQUIRE items and an in-depth explanation of each item.
- Please cite SQUIRE when it is used to write a manuscript.

**Text section and item name**	**Page/line no(s). info is located**
**Title and abstract**	
1. **Title**	1/1-2
Indicate that the manuscript concerns an initiative to improve healthcare (broadly defined to include the quality, safety, effectiveness, patient-centredness, timeliness, cost, efficiency and equity of healthcare).	
Response: Manuscript concerns an initiative to improve healthcare for forcibly displaced people.	Yes (explained left)
2. **Abstract**	1/1 - 2/9
a. Provide adequate information to aid in searching and indexing.	Yes
b. Summarise all key information from various sections of the text using the abstract format of the intended publication or a structured summary such as: background, local problem, methods, interventions, results, conclusions.	
Response: The abstract summarises all key information under headings of: objective, design, setting, participants, results, and conclusions.	Yes (explained left)
**Introduction: Why did you start?**	
3. **Problem description** - Nature and significance of the local problem.	2/32 - 3/8
4. **Available knowledge** - Summary of what is currently known about the problem, including relevant previous studies.	3/9-27
5. **Rationale** - Informal or formal frameworks, models, concepts and/or theories used to explain the problem, any reasons or assumptions that were used to develop the intervention(s) and reasons why the intervention(s) was expected to work	3/28 - 4/11
6. **Specific aims** - Purpose of the project and of this report.	4/12-33
**Methods: What did you do?**	
7. **Context** - Contextual elements considered important at the outset of introducing the intervention(s).	5/8-21

For peer review only

8. **Intervention(s)**	
a. Description of the intervention(s) in sufficient detail that others could reproduce it.	6/10-27
b. Specifics of the team involved in the work.	5/23 – 6/8
9. **Study of the intervention(s)**	
a. Approach chosen for assessing the impact of the intervention(s).	5/3-6
b. Approach used to establish whether the observed outcomes were due to the intervention(s).	6/24-29
10. **Measures**	
a. Measures chosen for studying processes and outcomes of the intervention(s), including rationale for choosing them, their operational definitions and their validity and reliability.	5/23 – 6/8
b. Description of the approach to the ongoing assessment of contextual elements that contributed to the success, failure, efficiency and cost.	6/10-24
c. Methods employed for assessing completeness and accuracy of data.	6/24-29
11. **Analysis**	
a. Qualitative and quantitative methods used to draw inferences from the data.	7/1-17
b. Methods for understanding variation within the data, including the effects of time as a variable.	6/11-13 and 7/9-13
12. **Ethical considerations** - Ethical aspects of implementing and studying the intervention(s) and how they were addressed, including, but not limited to, formal ethics review and potential conflict(s) of interest.	15/1-5
**Results: What did you find?**	
13. **Results**	
a. Initial steps of the intervention(s) and their evolution over time (e.g., time-line diagram, flow chart or table), including modifications made to the intervention during the project.	6/11-13
b. Details of the process measures and outcomes.	7/21-26 and 8/22-23
c. Contextual elements that interacted with the intervention(s).	7/26 – 8/19 and 8/25 – 11/28
d. Observed associations between outcomes, interventions and relevant contextual elements.	9/14-18; 9/29-33; 10/8-12; 11/9-11 and 11/16-18
e. Unintended consequences such as unexpected benefits, problems, failures or costs associated with the intervention(s).	11/28-32 and 9/17-18
f. Details about missing data.	12/13-15 and 12/34 – 13/2
**Discussion: What does it mean?**	
14. **Summary**	
a. Key findings, including relevance to the rationale and specific aims.	12/2-8; 12/18-23; 12/26-29 and 12/31 -13/2
b. Particular strengths of the project.	12/8-13; 12/15-17; 13/3-7 and 13/12-15
15. **Interpretation**	
a. Nature of the association between the intervention(s) and the outcomes.	13/7-15

For peer review only

b. Comparison of results with findings from other publications.	12/19-31 and 14/7-10
c. Impact of the project on people and systems.	13/12-15 and 14/14-22
d. Reasons for any differences between observed and anticipated outcomes, including the influence of context.	13/16-30 and 13/30-33
e. Costs and strategic trade-offs, including opportunity costs.	14/7-14
16. **Limitations**	
a. Limits to the generalisability of the work.	14/14-20
b. Factors that might have limited internal validity such as confounding, bias or imprecision in the design, methods, measurement or analysis.	13/16-17; 13/22-25 and 13/27-30
c. Efforts made to minimise and adjust for limitations.	13/30-33
**Conclusions**	
a. Usefulness of the work.	13/3-10
b. Sustainability.	13/10-15
c. Potential for spread to other contexts.	14/14-17
d. Implications for practice and for further study in the field.	14/17-20
e. Suggested next steps.	14/20-22
**Other information**	
18. **Funding** - Sources of funding that supported this work. Role, if any, of the funding organisation in the design, implementation, interpretation and reporting.	14/31-33

Ogrinc G, et al. BMJ Qual Saf 2015;0:1–7. doi:10.1136/bmjqs-2015-004411
Downloaded from http://qualitysafety.bmj.com/ on January 2, 2017

For peer review only

BMJ Open

Assessing the impact of artistic and cultural activities on the health and wellbeing of forcibly displaced people using participatory action research

Journal:	*BMJ Open*
Manuscript ID	bmjopen-2018-025465.R2
Article Type:	Research
Date Submitted by the Author:	17-Oct-2018

Complete List of Authors:	Clini, Clelia; Loughborough University - London, Institute for Media and Creative Industries<br>Thomson, Linda; University College London Division of Biosciences<br>Chatterjee, Helen; University College London, Division of Biosciences

**Primary Subject Heading**:	Qualitative research
Secondary Subject Heading:	Public health, Mental health
Keywords:	creative activities, forced displacement, participatory action research, post-traumatic stress disorder, refugees and asylum seekers

SCHOLARONE™ Manuscripts

Assessing the impact of artistic and cultural activities on the health and wellbeing of forcibly displaced people using participatory action research

Clelia Clini, Linda J Thomson, Helen J Chatterjee

Correspondence to: Helen J Chatterjee, UCL Division of Biosciences, 507B Darwin Building, University College London, London, WC1E 6BT, UK.
email h.chatterjee@ucl.ac.uk telephone +44(0)2031084104 fax +44(0)2076797193

Clelia Clini, Institute for Media and Creative Industries, Loughborough University London, UK

Linda J Thomson, UCL Division of Biosciences, University College London, London, UK

Key words: creative activities; forced displacement; participatory action research; post-traumatic stress disorder; refugees and asylum seekers;

Word count: 5315

Abstract

Objective: Drawing upon a growing body of research suggesting that taking part in artistic and cultural activities benefits health and wellbeing, the objective was to develop a participatory action research (PAR) method for assessing the impact of arts interventions on forcibly displaced people, and identify themes concerning perceived benefits of such programmes.

Design: A collaborative study following PAR principles of observation, focus groups and in-depth semi-structured interviews.

Setting. London-based charity working with asylum seekers and refugees.

Participants: An opportunity sample (n=31; 6m) participated in focus groups comprising refugees/asylum seekers (n=12; 2m), volunteers (n=4; 1m) and charity staff (n=15, 3m). A subset of these (n=17; 3m) participated in interviews comprising refugees/asylum seekers (n=7; 1m), volunteers (n=7; 1 m) and charity staff (n=3; 1m).

For peer review only - http://bmjopen.bmj.com/site/about/guidelines.xhtml

Results: Focus group findings showed that participants articulated the impact of creative activities around three main themes: skills, social engagement, and personal emotions that were explored during in-depth interviews. Thematic analysis of interviews was conducted in NVivo 11 and findings showed that artistic and cultural activities impacted positively by helping participants find a voice, create support networks, and learn practical skills useful in the labour market.

Conclusions: The study expanded on arts and wellbeing research by exploring effects of cultural and creative activities on the psychosocial wellbeing of refugees and asylum seekers. By focusing on the relationship between arts, wellbeing and forced displacement, the study was instrumental in actively trying to change the narrative surrounding refugees and asylum seekers, often depicted in negative terms in the public sphere.

Strengths and limitations of this study:

- Focus on collaborative research
- Different cultural and artistic activities studied
- Researcher spent five months in research setting
- Greater number of female than male participants
- Focus groups attracted relatively low numbers of refugees/asylum seekers

Introduction:

The recent All-Party Parliamentary Group on Arts Health and Wellbeing Inquiry Report shows a growing acknowledgement of the impact of arts and creative practices, stating ‘arts engagement has a beneficial effect upon health and wellbeing and therefore has a vital part to play in the public health arena’.[1 p11] Consensus among practitioners defines wellbeing as ‘the dynamic process that gives people a sense of how their lives are going through the interaction between their circumstances, activities and psychological resources or mental capital’.[2 p3] Rather than depending on a single factor, wellbeing is seen as the result of interplay between different elements, distinguishing personal wellbeing (positive emotion, life satisfaction, vitality, resilience, and self-esteem) from social wellbeing (supportive relationships, trust and belonging).[3] Other authors emphasise ‘resilience and flourishing, rather than just surviving’,[4 p243] a crucial point when investigating the situation of forcibly displaced people who often

For peer review only - http://bmjopen.bmj.com/site/about/guidelines.xhtml

survive in extreme conditions and whose experience is often discussed in terms of dehumanisation.[5, 6]

Research conducted in the field of forced displacement and mental health shows that ‘asylum seekers and displaced people report high rates of pre-migration trauma’,[6 p275] and typically display significant levels of depression, anxiety, post-traumatic stress disorder (PTSD) and non-affective psychoses.[7-11] Refugees and asylum seekers often encounter post-migration living difficulties, such as socio-economic disadvantage, employment barriers, social and emotional isolation, racism and hostility, experience of detention, and uncertainty related to the asylum application process.[7-9, 12] If, as authors suggest,[13-17] there is a strong link between mental health and socioeconomic conditions in terms of employability, income and housing, then the mental health of unemployed asylum seekers living on low incomes in poor quality housing is likely to be worse than that of the general population.

Arts-and-health practitioners believe that ‘aesthetics act upon our senses to make us feel more, hear more and see more than we otherwise might… feelings are intertwined with mental, physical, spiritual and social health’.[18 p4] Several studies explored the relationship between arts, health and wellbeing and a growing body of evidence suggests that cultural participation (including music, art making, theatre, dance, museum and heritage activities) enhances human health and wellbeing.[1, 19-27] Research conducted in museums-and-health, for example, suggests that ‘museum and art gallery encounters can help with a range of health issues, enhance wellbeing, and build social capital and resilience’.[27 p286] According to research conducted in Norway with over 50,000 adults,[28] participation in both receptive/passive and creative/active cultural activities was significantly associated with good health, life satisfaction, and low anxiety and depression. Findings demonstrated how manual creative practices such as knitting had a positive impact on people suffering from depression and post-traumatic stress disorder because ‘the movements involved in knitting are bilateral, rhythmic, repetitive, and automatic’.[28 p40] The authors attributed the positive mood of knitters to enhanced production of serotonin resulting from repeated movements, and that bilateral processes appeared to engage brain capacity and facilitate a meditative-like state more readily than unilateral ones. Although creative activities are seen to provide major benefits for refugees and asylum seekers, researchers have become ‘increasingly conscious of the value of recording and analysing what has been happening’.[8 p1]

For peer review only - http://bmjopen.bmj.com/site/about/guidelines.xhtml

The current study was conducted in the light of mental health and arts-in-health/museums-in-health evidence, and the large number of organisations that engage migrants, refugees and asylum seekers in the arts, with nearly 200 of these in the UK.[29] The current research was conducted at the Helen Bamber Foundation (HBF), a charity offering support to refugees and asylum seekers as victims of torture and human rights violations. HBF adopts a holistic approach to support its clients that includes cultural activities within a three-phase model of integrated care comprising stabilisation (medical and legal support), intervention (trauma-focused therapy and general psychological care), and integration (attending the creative, computing and English classes, and interacting with other clients). The model is based on the needs of refugee/asylum seekers who often require assistance on several fronts (e.g. psychological support might not be effective if a person does not have a place to sleep or lives in a dangerous situation, though giving people a place to sleep is not enough to overcome trauma). Their creative arts programme (CAP) led by volunteers includes art (painting and drawing), photography, textiles (dressmaking and knitting) and singing, and is attended by c.100 clients. The study took the view that arts participation would enhance the sense of wellbeing by allowing participants to form meaningful relationships based on trust and mutual support (improving social wellbeing) and enhance self-esteem and self-confidence (increasing personal wellbeing).

To provide voice and agency for participants, the study used a collaborative approach to explore client experiences of creative activities with HBF clients, staff and volunteers acting as co-researchers. The research was operationalised according to the principles of participatory action research (PAR)[30, 31] as a 'way of opening up space for dialogue and conversation',[32 p28] aiming to 'understand and also improve a particular situation',[32 p1] both pertinent aspects when working with displaced people. The PAR approach encourages an active contribution in the production of knowledge within a collaborative framework, emphasising 'equal partnerships',[33 p167] and the 'role of the participant in the design, implementation, and dissemination of the research'.[34 p78] The collaborative character of PAR is grounded in efforts to 'democratise the research process',[35 p3] so 'equal weight and consideration is given to the contributions of both the community and academic partners'[36 p170] again valuable for everyone involved but specifically refugees/asylum seekers. One of the pillars of PAR, especially when community-based is ownership.[37]

For peer review only - http://bmjopen.bmj.com/site/about/guidelines.xhtml

By offering equal weight to client contributions focusing on the relationship between forced displacement and arts participation, the study aimed to empower participants by developing their sense of ownership in the project. Through collaborative working between the charity, displaced people and academics, the purpose of the research was to co-develop a methodological approach to address the needs of refugees/asylum seekers and to co-produce interview questions that could be applied in UK and international settings. The objectives were to expand on arts-in-health and collaborative research approaches by exploring the benefits of engagement in cultural and creative activities on the health and wellbeing of forcibly displaced people and contribute to the current debate on migration and public health.

Method

Design

Qualitative research conducted comprised observation, focus groups and one-to-one semi-structured interviews with clients, volunteers and staff as co-researchers within a collaborative PAR approach.[38]

Participants

The study recruited an opportunity sample of volunteer participants through information leaflets and contact details in reception. Clients were approached by the researcher using two recruitment criteria: i) clients had received therapy for at least two years prior to the research and had entered the integration stage of the model of integrated care; this meant that though still vulnerable, clients had established a therapeutic relationship of trust enabling them to form safe relationships in the wider community and move forward with their lives; and ii) they had attended at least one of four CAP groups (art, photography, singing and textiles) for around two years. All participants spoke English sufficiently well to participate having attended English classes for about two years as part of integrated care. In total, 31(6m) participants volunteered for the study and attended focus group comprising 12(2m) refugees/asylum seekers, 4(1m) volunteers, and 15(3m) charity staff. Of these, 17(3m) participated in interviews comprising 7(1m) refugees/asylum seekers, 7(1m) volunteers, and 3(1m) staff.

For peer review only - http://bmjopen.bmj.com/site/about/guidelines.xhtml

Patient and public involvement

HBF staff were instrumental in the application for research funding and collaborated with the researcher to determine the study's objectives. HBF clients were involved in the recruitment process by recommending that other clients join them in the research, though the researcher ensured that they matched recruitment criteria (above). In keeping with PAR, research questions and outcome measures concerning the effects of cultural and creative activities on the psychosocial wellbeing of refugees/asylum seekers were determined by co-researchers (HFB clients, volunteers and staff) with differing involvement depending on their role at the charity. Focus groups and interviews gave voice to clients' priorities and preferences but, due to their vulnerability, no personal information was requested as the process of remembering could have been difficult.[7, 10] Although some participants mentioned personal issues and experiences, they were not encouraged to do so. Data generated by co-researcher involvement in the research collaboration informed focus groups, in turn focus group outcomes led to the development of questions for the in-depth interviews to follow. In addition to participation in focus groups and interviews, a core group of seven co-researchers (3 clients, 2 volunteers, 2 staff, with 1 male per category) were involved in design of the interview guides, discussion of emerging themes and dissemination of the research findings to other participants for verification and comments, in accordance with PAR principles.[33] The results of the research were fed back to everyone involved with HBF through on-going communication with the researcher, an internal report lodged at the charity, and an end-of-project event.

Data collection

Data collection carried out by the location-based researcher consisted of three stages: Stage 1 (months 1-2) participant observation; Stage 2 (months 3-4) focus groups; and Stage 3 (month 5) semi-structured interviews. In Stage 1, participant observation was used as a starting point 'for studying how organisations work, the roles played by different staff and the interaction between staff and clients'.[38 p32]. The researcher attended creative classes for clients and spent a day a week working from HBF (12-15 hours per week) to determine the nature of focus groups. In Stage 2, four focus groups were held, first and final with clients (n=8, n=4 respectively), second with volunteers (n=4), and third with staff (n=15). In the focus groups, participants were asked to discuss research questions (Table 1) involving ways of gathering information on the impact of

For peer review only - http://bmjopen.bmj.com/site/about/guidelines.xhtml

creative activities, and reasons for attending groups (or in the case of volunteers and staff, reasons for involvement). All focus groups were organised informally to facilitate development of discussion among participants and allow an exchange of experiences and ideas. Ideas that emerged during focus groups laid the basis for first drafts of interview questions, tailored to clients, volunteers or staff (Table 2). In line with the collaborative ethos, drafts were circulated to receive feedback from co-researchers prior to the interview stage, and volunteers and staff were consulted and asked to provide comments and suggestions for interview questions. For Stage 3, clients (n=7), volunteers (n=7) and staff (n=3) participated in interviews with the researcher using a semi-structured format to allow free expression within the research constraints and limit potential research bias.[39, 40]

INSERT TABLE 1 ABOUT HERE

INSERT TABLE 2 ABOUT HERE

Data analysis

A first informal thematic analysis was conducted by the researcher while writing field notes consisting of a chronicle of descriptive rather than analytic events both observed and provided by co-researchers as the ‘raw material of the research’.[30 p38] The process of transcribing notes was used to identify key points connected to the research question. This preliminary analysis informed focus group topics where points were discussed more in detail. After the first round of focus groups, all notes were compiled into a Word document and repeatedly read by the researcher and co-researchers to search for recurring topics and themes. Thematic analysis of focus group outcomes were explored in detail during interviews. All interviews were recorded and transcribed to become familiar with the data and begin the coding process [37]. All details in the transcripts were recorded verbatim (e.g. sighs, laughter, silences, and tears) as their exclusion could have changed the meaning expressed.[30] Transcriptions were uploaded into NVivo11 to produce nodes from coding-relevant concepts. NVivo’s word frequency query facilitated the search for key words (including stem words and synonyms) grouped under the same theme. The text search query provided a comprehensive analysis of data. After coding of data into initial themes,[37] a review of themes followed to refine the analysis.

For peer review only - http://bmjopen.bmj.com/site/about/guidelines.xhtml

Results

Focus Groups

Focus group data were analysed using thematic analysis (NVivo11). Findings showed consistency in the way all HBF participants (clients, volunteers and staff) articulated their reflections on the impact of creative activities. The benefits of creative activities were highlighted in clusters emerging from around three main overarching themes: 'social aspects/friendships', 'skills', and 'mood-personal sphere'; the latter sub-divided into 'brain' (creative thought processes), 'routine', self-expression' and 'confidence'. During focus groups, co-researchers were asked to write answers to the questions posed, and a collective discussion followed. Clients responded consistently about benefits of the creative programmes. One client listed 'Activities help my brain think about other stuff and keep me busy and relax. You can't think about bad things. To learn more skills. I can use skills to help others.' Staff commented on the mood enhancing benefits of CAP for people with PTSD; one staff member observed 'creative activities allow clients to momentarily leave out their problems or memories, by focusing on a particular activity they cannot think about anything else'. The focus group with HFB staff emphasised that being busy in the company of others allowed clients to feel safe and not to dwell upon their own situation. In concentrating on learning new skills, clients blocked out (albeit for short periods) memories of the past. Learning new or improving existing skills, the second theme to emerge during focus groups, appeared to be an important factor in improving self-esteem; another client noted that attending CAP 'Give me confidence. Make me feel good and useful. Change my mood. Learn new things and meet people. Improve my skills. Give me hope to lead a better life'. Furthermore, acquiring new skills appeared to change client perceptions of their status; as a female volunteer at HBF for two years suggested 'participants do not feel assessed as clients, they are just learners or artists. These groups paved the way for identifications different from being a victim, a refugee, or an asylum seeker'.

The third and final theme that emerged when discussing the benefits of CAP was its social aspect; it allowed people to meet others in similar situations and create friendships and/or a support network; as a client wrote 'Allows me to feel included. Safe environment. Improves my mood. Learn new skills. Gets me out of the house. Meet new people'. Staff too recognised the social aspect of activities as a major benefit, with one suggesting 'in these groups, clients learn to have balanced, reciprocal relationships, which they have not experienced before or for a long time given their experience of violence (trafficking). They provide clients with an alternative identity, i.e. they are not simply victims here, they are learners, artists, dressmakers, etc.' Similar themes emerged in the volunteer focus group during which a relatively

For peer review only - http://bmjopen.bmj.com/site/about/guidelines.xhtml

new male volunteer involved of four months was impressed by the solidarity between clients, emphasising the benefits of the 'feeling of belonging to a group' and 'strong support that people receive from other members'. Importantly, responses illustrated that focus group participants perceived their involvement as beneficial at multiple and overlapping levels. Enhancement of mood appeared to derive from not only meeting new people, forging meaningful relationships and feeling part of a group but also was connected to a growing self-confidence and self-esteem experienced as clients acquired new skills.

Interviews

During interviews, clients elaborated on practical and social skills, and commented on the positive effect of attendance on their mood and emotion:

Practical skills: Learning new practical skills or improving those previously learnt emerged strongly among asylum seekers without the right to work. As a client pointed out:

> *I have learned a lot of skills: I've learned sewing, now I'm learning art, I can do better, before I didn't know how to draw a person, to mix colours, you know such things... so you keep on learning, slowly by slowly, then in the end you find yourself, you're a professional, so that is very great... to me... I like to come, always and attend, and listen to what they tell me, and I do it.*

Or, as another client explained:

> *I've learned new skills [...] and you can never waste your time learning new skills. New skills always help you in your life, always. So, everything I've learned will help me. You think like 'film club, learning how to edit, etc.'. People pay people on YouTube to edit their films, and I'm, like, I could just do my own, if I wanted to have a YouTube channel, I can do my own.*

Skill improvement provided a sense of achievement and improved self-confidence, talking about learning new skills, one client observed:

> *When you are in this situation it feels like life has, in a way, stopped. And you can't do anything to change it. But by doing all this activity you feel, or I felt…it was like, you know, that I was learning something in my life, rather than just waiting.[…] For instance, if I go for a job somewhere, where you have to write what skills you have, so I could include all of this. I mean I don't have a certificate or, like, proper qualifications, but I've learned all*

For peer review only - http://bmjopen.bmj.com/site/about/guidelines.xhtml

those skills, so I feel like, it is, in a way, impressive? Because you feel like, you know, rather than just waiting and not doing anything, you have been learning.

The sense of self-improvement associated with learning skills was linked to the perception that by learning, clients were preparing themselves for a new life when they would be allowed to live and work in the UK, countering the perception that life for them had stopped and giving them something to look forward to. The process of skills learning was not simply a vertical one; peer learning was equally important, stressing the importance of social aspects.

Social skills: The opportunity to overcome social isolation and create a social network emerged as one of the benefits of creative engagement. As one client indicated:

The groups changed me because you know when I come, I feel lonely and doesn't have anybody. I was lonely I don't even know whether there will be a home from morning to live in. But when I started coming these groups, I told you I feel active, I feel coming to see people, that I speak to. I don't even know how to communicate with people before. I told you because of my coming here I now feel to communicate to people. Before I never communicate to people, I don't know how to do it. And I don't know how to play with people. But these groups make me now taught me: play with people, to meet me.

The client expressed the difficulties of establishing new relationships and the feeling that the group was a place in which they could regain this ability to connect with other people. The social aspect was an important point reiterated in all interviews; fellow clients were described as 'friends' but also as 'brothers and sisters'. Arts activities allowed clients to create a community characterised by solidarity, as one client explained:

If you're in a situation where you've been completely isolated from people for a while and you just don't know who to trust, or to be around people, it's one of those spaces where you can get to meet people, socialise, and actually make friends.[…] And these friendships do last. A life-time, because you understand what the person has gone through, you don't have to explain a lot, you know, they just know that you've been through something terrible.[…] So, it's easier when you have people here who just understand what you've possibly been through.

This remark connects with the observation that 'engagement in participative creative arts activities in communities can help to build social capital, address loneliness and social

For peer review only - http://bmjopen.bmj.com/site/about/guidelines.xhtml

isolation, and build personal confidence and a sense of empowerment'.[25 p32] The awareness of experiencing similar situations makes clients feel free to share their own experiences with one another but also not to, if they did not feel like opening up:

> *There is a sense of community: you know you go there, you know there are people like you in the same situation you're not going to be judged, so it's that sense of community: we know what's going on, we don't have to talk about it.[...] It's a distraction from immigration and we know we are all going through it, but we don't have to talk about it. There are other things going on in life, and we talked about, for example in the art group, the works we produced and what we could do, what we could achieve and get inspired by each other's work.*

Again, this remark introduced the third broad theme which emerged out of the interview analysis, which was the impact of creative arts on personal mood and emotion.

Mood and Emotion: Learning or improving skills while forging long-lasting relationships in a context of deep social isolation (outside of HBF), inevitably had a substantial impact on mood and emotion. In discussing this aspect of creative arts, interviewees focused particularly on how groups allowed them to have a routine, a 'luxury' one client said 'when you can't do nothing but waiting for the government to decide on your right to stay in the country'. Routine combined with skills learning/improving was thought to have a positive impact on clients' mental health; 'having something to do' and 'something to look forward to' were recurrent expressions during interviews. As one client noted:

> *I used to come here every day of my life: Monday, Tuesday, Wednesday... because I don't have anywhere to go, so I just come here, and I attached myself to the groups, we met, we talk, we... then I've gone to knit, I've gone to do computer, reading, many things I learnt here, so I just like it.*

Another client elaborated further:

> *It's something that I look forward to. One thing you have to bear in mind is that I am not working, and I am not studying, you know, so it was the only thing that I would look forward to because it was something to do, otherwise I would just be at home, doing nothing, you know, feeling very sorry for myself, getting upset all the time. I mean I still feel that way but at least there's something to look forward to, when you don't have hope or*

For peer review only - http://bmjopen.bmj.com/site/about/guidelines.xhtml

something to look forward to, it increases your depression levels, so for me it's helped me greatly.

This statement describes the social and emotional isolation as part of post-migration living difficulties. The routine of classes, together with an awareness that others in similar situations would attend, provided comfort to clients, countering feelings of isolation:

There is nothing else for you and it's also a way of, like, using your time wisely, 'cause at the end of the day most of the time you're just sitting down doing nothing, and doing nothing slowly begins to affect your mind, your brain, you know you become lazy, you know you literally are just in a four-squared room[…]

The benefits of a routine were increased by inclusion of creative activities that according to clients and volunteers, had a positive impact on mood because they exercised the brain and allowed clients to find a new language to express themselves. As a client stated:

[Attending creative groups] gives you a lot of confidence, you know. And it allows your brain to think outside of the box because I think we are just in that box and... but when you attend these classes, your mind, you know, opened and you learn new things and you want to then learn other things as well.

This comment highlights an important link between arts and feelings; as another stated:

It is more of expressing, more of letting go, it's like getting a spirit out of you, and you don't have necessarily have to tell someone 'I've done this because of that and that, and this', you know. Because sometimes you just don't find the voice to talk about it and, I, as a person am really shy and, you know, I feel easily embarrassed, you know.[…] So... that's why I'm into arts, yes I'm doing arts really.

Clients agreed that the possibility of self-expressing without having to articulate their feelings, helped them grow emotionally and gain confidence. It was not only clients who benefited from CAP; findings also pointed to the positive impact on volunteers, a point stressed by all volunteers when discussing personal experiences.

Discussion

Creative and cultural activities were observed over a sustained period of five months. Clients participating in the activities had reached the integration stage of the model of integrated care consequently had been taking part in CAP activities for two plus years prior to the research.

For peer review only - http://bmjopen.bmj.com/site/about/guidelines.xhtml

Activities provided refugees and asylum seekers with new skills, including practical and technical skills, and social and life skills involving language acquisition obtained partly through informal peer learning and mutual support. Learning new skills contributed to the sense of wellbeing and empowerment experienced by HBF clients. The time spent by the researcher at the HBF was vital for familiarisation with the context, getting to know people involved in the organisation, and explaining the research to recruit participants from among clients, volunteers and staff. The fact that several clients attended more than one group per week (2-4) made it possible to forge trusting relationships and recruit co-researchers, allowing them to feel comfortable in speaking their mind. A possible issue here was that the number of groups attended by clients was not accounted for, so it was not evident as to whether increased participation furthered benefits at a higher/faster level. Once recruited, participants remained with the study in their co-researcher roles and continued to attend activities on a weekly basis for the five months (except for rare absences due to illness).

Activities appeared to positively enhance mood and emotion both for clients and volunteers facilitating CAP groups. Although passive participation was not compared,[28] active and creative participation in the groups specifically benefited the clients. A key reason was that asylum seeker status in the UK would not have permitted alternative occupation or employment, so clients might not have otherwise left their homes or met people on a regular basis. In keeping with previous research, creative and cultural participation led to improved social health,[18] and aligned with a growing body of evidence[1] to suggest that creative activities enhanced mental wellbeing,[19-27] helping clients to develop self-confidence and resilience.[21] Client preferences for activities indicated that singing was their favourite group, followed by photography, art and textiles. Singing was regarded as beneficial because it allowed people to meet socially and work collaboratively, aspects considered important in countering loneliness. Findings aligned with a recent qualitative study indicating that choral singing promoted improvements in social, emotional, physical, and cognitive functioning and that benefits were experienced similarly irrespective of age, gender, nationality.[41] In the current study, photography, art and textiles were praised by clients for the skills taught and although social aspects were relevant, skills that allowed participants to continue activities at home (e.g. sewing, drawing) were highly valued. Interviewed staff were asked to comment on the popularity of

For peer review only - http://bmjopen.bmj.com/site/about/guidelines.xhtml

activities, and although the order was the same as that of clients, they were not asked their opinion on which were most beneficial.

The current study bridged the gap between two research disciplines: arts-in-health and forced displacement and mental health. It contributed to existing literature by demonstrating beneficial effects of creative activities on the wellbeing of refugees/asylum seekers and showed that PAR was an appropriate and 'democratic' means of collaboration between displaced communities and academics.[40 p3] A core element of PAR is that research should be meaningful and have tangible outcomes for co-researchers to bring about significant change within their community or society at large. PAR contrasts with traditional methods where 'academics benefit from the research, but often the people involved hear very little if anything from the researcher again'.[33 p78] In the current study, client involvement in the research and their decision to organise a public exhibition to showcase their artwork was a means of interacting with wider society and actively re-writing the narrative surrounding refugees and asylum seekers, often depicted in negative terms in the public sphere.[42]

Adopting a PAR approach is important when working with immigrant communities as it ensures that the research question is of 'genuine importance',[34 p118] and facilitates translation of research findings into 'actions that will benefit the community'.[34 p119] PAR was particularly useful when conducting research with refugees/asylum seekers with PTSD and other mental health issues because the process of involvement as co-researchers countered client isolation and built social capital through regular meetings. In helping to interpret findings, clients acquired research methods skills (e.g. drafting interview guides, conducting focus groups, discussing data) and organisational abilities (event/exhibition). Participating in the research and disseminating findings gave clients a sense of agency and ownership of the project that enhanced self-confidence and feelings of empowerment, one of the pillars of PAR especially when community based.[35 p2] Participation provided a positive means of expression countering their refugee/asylum seeker status that otherwise, rendered them effectively powerless.

In terms of study weaknesses, the greater number of female than male participants was probably due to the fact that most creative groups were led by female volunteers (2m, 8f). Most clients attending these groups were also female except for singing, where numbers were approximately equal (although only one male choir member took part in a focus group). Given the nature of the singing activity, conversation was limited to the first minutes before arrival;

For peer review only - http://bmjopen.bmj.com/site/about/guidelines.xhtml

once rehearsals had started it was difficult for the researcher to form relationships with clients. It was also possible that some activities were seen by clients as gender-neutral (e.g. photography or singing) but others such as working with fabrics and soft materials (e.g. wool and cloth) may have been seen as a predominantly female activity; male participants might have been more comfortable working with harder materials (e.g. wood and metal). It was generally more difficult to engage male participants in the research; a possible reason for this was female clients might have found it easier to relate to the female researcher. A further weakness was low attendance for focus groups possibly due to difficulties of organising these at convenient times for everyone, given changing client circumstances (e.g. interviews for benefits, housing etc.). More clients expressed an interest than those who participated, nevertheless were happy to informally discuss topics debated. Others wanted to be kept in the loop without participating in focus groups and interviews, so research methods were discussed informally during meetings between researcher and clients.

Conclusions

This study expanded on arts-in-health and collaborative research approaches by exploring the effects of cultural and creative activities on the health and wellbeing of refugees and asylum seekers. Creative activities at HBF provided opportunities for clients to socialise and develop new relationships based on respect and mutual recognition. In turn, these opportunities countered loneliness and allowed refugees/asylum seekers to develop a sense of belonging in a safe space. The opportunities further supported freedom of expression and the possibility of developing a new identity to oppose the negative stereotype associated with refugee/asylum seeker status,[42] leading to sustained improvements in positive mood and self-confidence, and countering anxiety, depression and other mental health issues. By focusing on the relationship between arts, wellbeing and forced displacement, and in keeping with PAR, the study actively changed the narrative surrounding refugees/asylum seekers and gave an understanding of how to improve conditions for forcibly displaced people through opportunities for social connectivity, creative thinking and employment skills. It is recommended that other refugee/asylum seeker organisations utilise the interview questions generated by the study, or develop their own following PAR guidelines, to appraise and improve migrant provision particularly in terms of up-skilling peer learning. If extended across the UK, programmes of creative activities for migrants

For peer review only - http://bmjopen.bmj.com/site/about/guidelines.xhtml

could reduce pressure on health and social care services and help to address the social determinants of health inequality.[16] Research outputs have wider implications for policy development, for example by the United Nations Refugee Agency, World Health Organisation, and other government and non-government agencies.

Contributors

CC, LT and HC made substantial contributions to the conception and design of the work; drafting and critically revising the work for intellectual content; and the final approval of the version published. CC conducted the acquisition, analysis and interpretation of data for the work. All authors agree to be accountable for all aspects of the work. We would like to thank the clients, volunteers and staff who were co-researchers in the project.

Funding

The work was supported by the Global Challenges Research Fund (GCRF) Grant reference: ES/P003818/1 PI: HC

Ethics

The study gained approval from UCL Ethics Committee (Ethics Application 4526/002 Co-developing a method for assessing the psychosocial impact of cultural interventions with displaced people: Towards an integrated care framework) to carry out the research with potentially vulnerable participants.

Competing interests

The authors have no competing interests.

Data sharing statement

Due to the confidential nature of the human participant data, it will not be freely available via free access databases. Any request for data should be addressed to the corresponding author.

References

For peer review only - http://bmjopen.bmj.com/site/about/guidelines.xhtml

1. All-Party Parliamentary Group on Arts, Health and Wellbeing (APPGAHW). Creative Health: The Arts for Health and Wellbeing. Inquiry Report. 2017; http://www.artshealthandwellbeing.org.uk/appg-inquiry/
2. New Economics Foundation (NEF). 2009. National accounts of wellbeing: What is wellbeing? NEF. http://www.nationalaccountsofwellbeing.org/learn/what-is-well-being.html
3. New Economics Foundation (NEF). National Accounts of Well-being: Bringing Real Wealth onto the Balance Sheet. 2009. https://neweconomics.org/uploads/files/2027fb05fed1554aea_uim6vd4c5.pdf
4. Ander E, Thomson LJ, Noble G, Lanceley A, Menon U, Chatterjee H. Generic well-being outcomes: Towards a conceptual framework for well-being outcomes in museums. Int J Museum Manage Curator 2011; 26:237–259. doi:10.1080/09647775.2011.585798
5. Crawley H, Düvell F, Jones K, McMahon S, Sigona, N. Destination Europe? Understanding the dynamics and drivers of Mediterranean migration in 2015. MEDMIG Final Report 2016. www.medmig.info/research-brief-destination-europe.pdf
6. Vaughan-Williams N. “We are *not* animals!” Humanitarian border security and zoopolitical spaces in Europe. Political Geography 2015;45:1–10. doi:10.1016/j.polgeo.2014.09.009
7. Katona C. Non-affective psychosis in refugees. Br Med J 2016;352:i1279. doi:10.1136/bmj.i1279
8. Robjant K, Robbins I, Senior V. Psychological distress amongst immigration detainees: A cross-sectional questionnaire study. Br J Clin Psychol 2009;48:275–286. doi:10.1348/014466508X397007
9. Alpak G, Unal A, Bulbul F, Sagaltici E, Bez Y, Altindag A et al. Post-traumatic stress disorder among Syrian refugees in Turkey: A cross-sectional study. Int J Psychol Clinic Practice 2015;19:45–50. doi:10.3109/13651501.2014.961930
10. Schubert CC, Punamäki R. Mental health among torture survivors: cultural background, refugee status and gender. Nordic J Psychol 2011;65:175–182. doi:10.3109/08039488.2010.514943
11. Silove D, Sinnerbrink I, Field A, Manicavasagar V, Steel Z. Anxiety, depression and PTSD in asylum-seekers: associations with pre-migration trauma and post-migration stressors. Brit J Psych 1997;170:351–357. doi:10.1192/bjp.170.4.351 doi:10.1348/014466508X397007

For peer review only - http://bmjopen.bmj.com/site/about/guidelines.xhtml

12. Aragona M, Pucci D, Mazzetti M, Geraci S. Post-migration living difficulties as a significant risk factor for PTSD in immigrants: a primary care study. Italian J Public Health 2012;9:e7525;1–8. doi:10.2427/7525

13. Allen J, Allen M. The social determinants of health, empowerment and participation. In S Clift, P Camic (Eds.), Oxford Textbook of Creative Arts, Health, and Wellbeing: International Perspectives on Practice, Policy and Research. New York, Oxford: Oxford University Press; 2016, p27–34.

14. Fisher M. Why mental health is a political issue. The Guardian, July 16, 2012. https://www.theguardian.com/commentisfree/2012/jul/16/mental-health-political-issue

15. Griffin J. The Lonely Society. London: The Mental Health Foundation. https://www.mentalhealth.org.uk/sites/default/files/the_lonely_society_report.pdf

16. Marmot M, Allen J, Goldblatt P, Boyce T, McNeish D, Grady M et al. Fair Society, Healthy Lives: The Marmot Review. 2010; http://www.instituteofhealthequity.org/resources-reports/fair-society-healthy-lives-the-marmot-review/fair-society-healthy-lives-full-report-pdf

17. Paul K, Moser K. Unemployment impairs mental health: Meta-analyses. J Vocational Behavior 2009;74:264-282. doi:10.1016/j.jvb.2009.01.001

18. Prior RW. Editorial. J Applied Arts Health 2009;1:3–6.

19. Bygren LO, Johansson S, Konlaan BB, Grjibovski AM, Wilkinson AV, Sjöström M. Attending cultural events and cancer mortality: A Swedish cohort study. Arts Health: Int J Res Pol Pract 2009;1:64–73. doi:10.1080/17533010802528058

20. Camic P, Chatterjee H. Museums and art galleries as partners for public health interventions. Perspect Public Health 2013;133:66–77.

21. Chatterjee H. Museums and art galleries as settings for public health interventions. In S Clift, P Camic (Eds.), Oxford Textbook of Creative Arts, Health, and Wellbeing: International Perspectives on Practice, Policy and Research. New York, Oxford: Oxford University Press 2016; p.281–289.

22. Clift S, Skingley A, Coulton S, Rodriguez J. The effectiveness and cost-effectiveness of a participative community singing programme as a health promotion initiative for older people: protocol for a randomised controlled trial. BMC Public Health 2011;11:1–6. doi:10.1186/1471-2458-11-142

For peer review only

For peer review only - http://bmjopen.bmj.com/site/about/guidelines.xhtml

23. Clift S, Camic P. Oxford Textbook of Creative Arts, Health, and Wellbeing: International Perspectives on Practice, Policy and Research. New York, Oxford: Oxford University Press, 2016.

24. Konlaan BB, Bygren LO, Johansson S. Visiting the cinema, concerts, museums or art exhibitions as determinant of survival: a Swedish fourteen-year cohort follow-up. Scand J Public Health 2000;28:174–178. doi:10.1177/14034948000280030501

25. Staricoff RL. Arts in health: A review of the medical literature. London: Arts Council England, Research Report 2004;36:1–91. http://www.artsandhealth.ie/wp-content/uploads/2011/08/AHReview-of-Medical-Literature1.pdf

26. Staricoff RL. Arts in health: The value of evaluation. Perspect Public Health 2006;126:116–120. http://journals.sagepub.com/doi/pdf/10.1177/1466424006064300

27. Chatterjee HJ, Noble G. Museums, Health and Wellbeing. Farnham: Ashgate, 2013.

28. Cuypers K, Krokstad S, Holmen TL, Knudtsen MS, Bygren LO, Holmen J. Patterns of receptive and creative cultural activities and their association with perceived health, anxiety, depression and satisfaction with life among adults: the HUNT study, Norway. J Epidemiol Community Health 2012;66:698–703. https://jech.bmj.com/content/jech/66/8/698.full.pdf

29. Kidd B, Zahir S, Khan S. Arts and Refugees: History, Impact and Future. London: Arts Council England, 2008. https://baringfoundation.org.uk/wp-content/uploads/2014/10/ArtsandRefugees.pdf

30. Kemmis S, McTaggart R, Nixon R. The Action Research Planner: Doing Critical Participatory Action Research. Singapore: Springer 2014.

31. Bradbury H. Introduction: How to situate and define action research. In H Bradbury (Ed.). The Sage Handbook of Action Research, London: Sage 2015. p.1–9.

32. Nicolaidis C, Raymaker DM. Community based participatory research with communities defined by race, ethnicity, and disability: Translating theory to practice. In H Bradbury (Ed.), The Sage Handbook of Action Research. London: Sage 2015. p.167–178.

33. Daykin N, Stickley T. The role of qualitative research in arts and health. In S Clift, P Camic (Eds.), Oxford Textbook of Creative Arts, Health, and Wellbeing: International Perspectives on Practice, Policy and Research. New York, Oxford: Oxford University Press 2016, p.73–82.

For peer review only - http://bmjopen.bmj.com/site/about/guidelines.xhtml

34. Vaughn LM, Jacquez F. Community-Based Participatory Research Studies Involving Immigrants. In S Coughlin, SA Smith, ME Fernandez (Eds.), Handbook of Community-Based Participatory Research. New York, Oxford: Oxford University Press, 2017. p.115–130.
35. Blumenthal DS. Is Community-based participatory research possible? Am J Prev Med. 2011;40:386–389.
36. Braun V, Clarke V. Using thematic analysis in psychology. Qual Res Psychol 2006;3:77–101.
37. Pope C, Mays N. Qualitative Research in Health Care, 3rd Edition. Oxford: Blackwell, 2006.
38. Pope C, Ziebland S, Mays N. Analysing Qualitative Data. In C Pope, N Mays. Qualitative Research in Health Care, 3rd Edition. Oxford: Blackwell. 2006. p.63–81.
39. Kemmis S, McTaggart R, Nixon R. The Action Research Planner: Doing Critical Participatory Action Research. Singapore: Springer 2014.
40. Bradbury H. Introduction: How to Situate and Define Action Research. In H Bradbury (Ed). The Sage Handbook of Action Research, London: Sage 2015. p.1–9.
41. Livesey L. Morrison I. Clift S. Camic P. Benefits of choral singing for social and mental wellbeing: Qualitative findings from a cross-national survey of choir members. J Pub Mental Health 2012;11:10–26.
42. Khosravi S. 'Illegal' Traveller: An Auto-ethnography of Borders. Basingstoke: Palgrave MacMillan 2010.

Table 1. Focus group questions

Clients:<br>1. Why do you like the HBF creative arts programme?<br>2. How would you gather information or evidence about the benefits of artistic activities?<br>3. Which creative arts classes have a positive impact on the lives of refugees?<br>Circle the option(s) that you think work best or add another one.<br>Arts and crafts Drawing Films Knitting Photography Singing Textiles.
Volunteers:<br>1. Why do you volunteer at the HBF?<br>2. How do you think your group benefits clients?<br>3. How would you collect evidence on the effects of participating in artistic activities?

For peer review only

Staff:

1. Why do you recommend clients to attend Creative Arts groups?
2. What do you think are the benefits of attending arts groups?
3. How would you collect evidence on the effects of participating in artistic activities?

Table 2. Interview questions

Clients:

1. How long have you been attending the CAP classes for?
2. Which groups do you attend?
3. Why did you choose this/these groups(s)?
4. What do you like the most about these groups?
5. Is there anything about the groups that you find difficult?
6. Anything you would like to change?
7. Is there a group you prefer? Which one? Why?
8. How do you feel when you attend this group?
9. Do you think there are benefits of attending these groups? If so what are they?
10. During our previous meeting, it emerged that participants feel that groups allow them to learn new or improve already acquired skills. Do you agree? Have you learnt any new skill? And what do you think about the possibility of learning new skills? (How does that impact your life)
11. Another element which emerged during our meeting is that people enjoy the social aspect of these groups: meeting people and finding new friends. What do you think about this? [supporting questions: do you like being around other people? Why? What happens when you attend a session? Have you made new friends? How do you feel about that?]
12. Among all of the reasons why you enjoy attending the creative arts group, which one is the one that you feel strongest about?
13. Why do you keep attending?

For peer review only

For peer review only - http://bmjopen.bmj.com/site/about/guidelines.xhtml

14. If you could compare the way you were feeling before joining any of these activities and the way you feel now, would you say you feel any different? Explain
15. Creative activities of course are part of the support that HB offers to its clients. Do you think that you would feel the same about yourself today even without attending these creative activities groups? Why?
16. Do you think that taking part in these creative activities has had any influence on the way you see yourself? And the way you see yourself in London?
17. If you were to recommend someone to join a group, what would you say?

For peer review only

For peer review only - http://bmjopen.bmj.com/site/about/guidelines.xhtml

Volunteers:

1. What do you teach?
2. How long have you been volunteering for?
3. Why did you decide to volunteer?
4. How did you learn about the Helen Bamber Foundation?
5. What do you think your group offers clients?
6. What are the main challenges you face/have faced as a volunteer?
7. Do you find it difficult to engage clients in the activity you coordinate? What do you think clients like about the way you (and your colleagues) handle the sessions? Is there anything that clients don't like?
8. How would you describe a typical session?
9. What do you think people like the most about your group?
10. Do you ever discuss emotions with clients? Or their personal situation?
11. Look at the notion of wellbeing defined by NEF (New Economics Foundation):

Wellbeing as 'the dynamic process that gives people a sense of how their lives are going through the interaction between their circumstances, activities and psychological resources or 'mental capital.' (NEF 2008 p3).

According to the NEF, there are different components to wellbeing, such as:
'Personal wellbeing (emotional wellbeing, satisfying life, vitality, resilience, self-esteem and positive functioning) and social wellbeing, including supporting relationships and trust and belonging.' (NEF 2009).

12. Do you think that the activity you lead has any impact on the wellbeing of clients? Elaborate
13. If you were asked to recruit people for your group, what would you say to convince them to join?
14. Would you use different words to promote your group with men and women? Why?
15. Do you think that your group makes a difference in the lives of clients? How? Why?

For peer review only - http://bmjopen.bmj.com/site/about/guidelines.xhtml

16. And what about your own life? What is the impact of your volunteer activity on your own life?

Staff:

1. How long have you been working at HB for?
2. Why did you decide to work here?
3. What is your role?
4. As part you work, do you have any direct contact with clients?
5. Could you say a few words on your relationship with clients?
6. How would you describe your own experience at HB so far?
7. What are the main challenges that you face/have faced in your position?
8. Do you think that your own gender influences the ways in which clients relate to you?
9. In your position, do you discuss clients' personal situations and feelings?
10. If so, do you think that the cultural background of clients affects the way in which you discuss emotions? If it does, how and why?
11. What are the main challenges of discussing emotions with clients?
12. Could you say a few words on the Model of Integrated Care? Do you think it works? And if so, why?
13. Do you recommend clients to attend any of the CAP groups? What do you say in this case?
14. What role do you think cultural and creative activities play in the recovery of clients?
15. Are there any particular activities that, in your opinion, are more popular with clients?

For peer review only

16. Would you say that the programme makes a difference in the lives of clients? If yes, please elaborate.
17. Have you witnessed clients benefiting from their own participation in the creative arts programmes? If yes, in what ways?

For peer review only

For peer review only - http://bmjopen.bmj.com/site/about/guidelines.xhtml

Research and reporting methodology
Revised **Standards for Quality Improvement Reporting Excellence** (**SQUIRE 2.0**) publication guidelines

Notes to authors

- The SQUIRE guidelines provide a framework for reporting new knowledge about how to improve healthcare.
- The SQUIRE guidelines are intended for reports that describe system level work to improve the quality, safety and value of healthcare, and used methods to establish that observed outcomes were due to the intervention(s).
- A range of approaches exists for improving healthcare. SQUIRE may be adapted for reporting any of these.
- Authors should consider every SQUIRE item, but it may be inappropriate or unnecessary to include every SQUIRE element in a particular manuscript.
- The SQUIRE glossary contains definitions of many of the key words in SQUIRE.
- The explanation and elaboration document provides specific examples of well-written SQUIRE items and an in-depth explanation of each item.
- Please cite SQUIRE when it is used to write a manuscript.

**Text section and item name**	**Page/line no(s). info is located**
**Title and abstract**	
1. **Title**	1/1-1/2
Indicate that the manuscript concerns an initiative to improve healthcare (broadly defined to include the quality, safety, effectiveness, patient-centredness, timeliness, cost, efficiency and equity of healthcare).	
Manuscript concerns an initiative to improve healthcare for forcibly displaced people.	
2. **Abstract**	1/21-2/9
a. Provide adequate information to aid in searching and indexing.	1/21-2/9
b. Summarise all key information from various sections of the text using the abstract format of the intended publication or a structured summary such as: background, local problem, methods, interventions, results, conclusions.	1/21-2/9
The structure abstract summarises all key information under headings of: objective, design, setting, participants, results, and conclusions.	1/21-2/9
**Introduction: Why did you start?**	
3. **Problem description** - Nature and significance of the local problem.	2/32-3/8 and 3/28-4/11
4. **Available knowledge** - Summary of what is currently known about the problem, including relevant previous studies.	3/9-3/27
5. **Rationale** - Informal or formal frameworks, models, concepts and/or theories used to explain the problem, any reasons or assumptions that were used to develop the intervention(s) and reasons why the intervention(s) was expected to work	2/19-2/31 and 4/12-4/24
6. **Specific aims** - Purpose of the project and of this report.	4/25-4/33
**Methods: What did you do?**	
7. **Context** - Contextual elements considered important at the outset of introducing the intervention(s).	5/4-5/6

8. **Intervention(s)**	
a. Description of the intervention(s) in sufficient detail that others could reproduce it.	5/9-5/21
b. Specifics of the team involved in the work.	5/24-5/27
9. **Study of the intervention(s)**	
a. Approach chosen for assessing the impact of the intervention(s).	5/27-6/8<br>6/11-6/23
b. Approach used to establish whether the observed outcomes were due to the intervention(s).	6/23-6/27
10. **Measures**	
a. Measures chosen for studying processes and outcomes of the intervention(s), including rationale for choosing them, their operational definitions and their validity and reliability.	6/27-6/29
b. Description of the approach to the ongoing assessment of contextual elements that contributed to the success, failure, efficiency and cost.	7/2-7/11
c. Methods employed for assessing completeness and accuracy of data.	7/11-7/17
11. **Analysis**	
a. Qualitative and quantitative methods used to draw inferences from the data.	7/2-7/10
b. Methods for understanding variation within the data, including the effects of time as a variable.	6/14-6/17 and<br>7/10-7/17
12. **Ethical considerations** - Ethical aspects of implementing and studying the intervention(s) and how they were addressed, including, but not limited to, formal ethics review and potential conflict(s) of interest.	15/2-15/5
**Results: What did you find?**	
13. **Results**	
a. Initial steps of the intervention(s) and their evolution over time (eg, time-line diagram, flow chart or table), including modifications made to the intervention during the project.	5/3-5/21
b. Details of the process measures and outcomes.	6/10-7/17
c. Contextual elements that interacted with the intervention(s).	5/23-5/31 and<br>5/34-6/8
d. Observed associations between outcomes, interventions and relevant contextual elements.	7/19-7/33,<br>8/2-8/5,<br>8/10-8/21 and<br>8/26-11/32
e. Unintended consequences such as unexpected benefits, problems, failures or costs associated with the intervention(s).	7/33-8/2,<br>8/6-8/9 and<br>8/32-8/34
f. Details about missing data.	5/31-5/34
**Discussion: What does it mean?**	
14. **Summary**	
a. Key findings, including relevance to the rationale and specific aims.	12/2-12/8
b. Particular strengths of the project.	12/8-12/13 and<br>12/15-12/17
15. **Interpretation**	
a. Nature of the association between the intervention(s) and the outcomes.	12/18-12/23
b. Comparison of results with findings from other publications.	12/23-12/31

For peer review only

c. Impact of the project on people and systems.	13/3-13/17
d. Reasons for any differences between observed and anticipated outcomes, including the influence of context.	12/13-12/15 and 12/31-13/2
e. Costs and strategic trade-offs, including opportunity costs.	13/17-13/27
16. **Limitations**	
a. Limits to the generalisability of the work.	13/28-13/31
b. Factors that might have limited internal validity such as confounding, bias or imprecision in the design, methods, measurement or analysis.	14/32-14/8
c. Efforts made to minimise and adjust for limitations.	14/8-14/11
**Conclusions**	
a. Usefulness of the work.	14/14-14/19
b. Sustainability.	14/19-14/22
c. Potential for spread to other contexts.	14/22-14/26
d. Implications for practice and for further study in the field.	14/26-14/29
e. Suggested next steps.	14/29-14/34
**Other information**	
18. **Funding** - Sources of funding that supported this work. Role, if any, of the funding organisation in the design, implementation, interpretation and reporting.	15/9-15/11

Ogrinc G, et al. BMJ Qual Saf 2015;0:1–7. doi:10.1136/bmjqs-2015-004411
Downloaded from http://qualitysafety.bmj.com/ on January 2, 2017

For peer review only

BMJ Open

Assessing the impact of artistic and cultural activities on the health and wellbeing of forcibly displaced people using participatory action research

Journal:	*BMJ Open*
Manuscript ID	bmjopen-2018-025465.R3
Article Type:	Research
Date Submitted by the Author:	14-Dec-2018
Complete List of Authors:	Clini, Clelia; Loughborough University - London, Institute for Media and Creative Industries<br>Thomson, Linda; University College London Division of Biosciences<br>Chatterjee, Helen; University College London, Division of Biosciences
**Primary Subject Heading**:	Qualitative research
Secondary Subject Heading:	Public health, Mental health
Keywords:	creative activities, forced displacement, participatory action research, post-traumatic stress disorder, refugees and asylum seekers

SCHOLARONE™
Manuscripts

Assessing the impact of artistic and cultural activities on the health and wellbeing of forcibly displaced people using participatory action research

Clelia Clini, Linda J Thomson, Helen J Chatterjee

Correspondence to: Helen J Chatterjee, UCL Division of Biosciences, 507B Darwin Building, University College London, London, WC1E 6BT, UK.
email h.chatterjee@ucl.ac.uk telephone +44(0)2031084104 fax +44(0)2076797193

Clelia Clini, Institute for Media and Creative Industries, Loughborough University London, UK

Linda J Thomson, UCL Division of Biosciences, University College London, London, UK

Key words: creative activities; forced displacement; participatory action research; post-traumatic stress disorder; refugees and asylum seekers;

Word count: 5342

Abstract

Objective: Drawing upon a growing body of research suggesting that taking part in artistic and cultural activities benefits health and wellbeing, the objective was to develop a participatory action research (PAR) method for assessing the impact of arts interventions on forcibly displaced people, and identify themes concerning perceived benefits of such programmes.

Design: A collaborative study following PAR principles of observation, focus groups and in-depth semi-structured interviews.

Setting. London-based charity working with asylum seekers and refugees.

Participants: An opportunity sample (n=31; 6m) participated in focus groups comprising refugees/asylum seekers (n=12; 2m), volunteers (n=4; 1m) and charity staff (n=15, 3m). A subset of these (n=17; 3m) participated in interviews comprising refugees/asylum seekers (n=7; 1m), volunteers (n=7; 1 m) and charity staff (n=3; 1m).

Results: Focus group findings showed that participants articulated the impact of creative activities around three main themes: skills, social engagement, and personal emotions that

For peer review only - http://bmjopen.bmj.com/site/about/guidelines.xhtml

were explored during in-depth interviews. Thematic analysis of interviews was conducted in NVivo 11 and findings showed that artistic and cultural activities impacted positively by helping participants find a voice, create support networks, and learn practical skills useful in the labour market.

Conclusions: The study expanded on arts and wellbeing research by exploring effects of cultural and creative activities on the psychosocial wellbeing of refugees and asylum seekers. By focusing on the relationship between arts, wellbeing and forced displacement, the study was instrumental in actively trying to change the narrative surrounding refugees and asylum seekers, often depicted in negative terms in the public sphere.

Strengths and limitations of this study:

- Focus on collaborative research
- Different cultural and artistic activities studied
- Researcher spent five months in research setting
- Greater number of female than male participants
- Focus groups attracted relatively low numbers of refugees/asylum seekers

Introduction:

The recent All-Party Parliamentary Group on Arts Health and Wellbeing Inquiry Report shows a growing acknowledgement of the impact of arts and creative practices, stating 'arts engagement has a beneficial effect upon health and wellbeing and therefore has a vital part to play in the public health arena'.[1 p11] Consensus among practitioners defines wellbeing as 'the dynamic process that gives people a sense of how their lives are going through the interaction between their circumstances, activities and psychological resources or mental capital'.[2 p3] Rather than depending on a single factor, wellbeing is seen as the result of interplay between different elements, distinguishing personal wellbeing (positive emotion, life satisfaction, vitality, resilience, and self-esteem) from social wellbeing (supportive relationships, trust and belonging).[3] Other authors emphasise 'resilience and flourishing, rather than just surviving',[4 p243] a crucial point when investigating the situation of forcibly displaced people who often survive in extreme conditions and whose experience is often discussed in terms of dehumanisation.[5, 6]

Research conducted in the field of forced displacement and mental health shows that 'asylum seekers and displaced people report high rates of pre-migration trauma',[6 p275] and

For peer review only - http://bmjopen.bmj.com/site/about/guidelines.xhtml

typically display significant levels of depression, anxiety, post-traumatic stress disorder (PTSD) and non-affective psychoses.[7-11] Refugees and asylum seekers often encounter post-migration living difficulties, such as socio-economic disadvantage, employment barriers, social and emotional isolation, racism and hostility, experience of detention, and uncertainty related to the asylum application process.[7-9, 12] If, as authors suggest,[13-17] there is a strong link between mental health and socioeconomic conditions in terms of employability, income and housing, then the mental health of unemployed asylum seekers living on low incomes in poor quality housing is likely to be worse than that of the general population.

Arts-and-health practitioners believe that 'aesthetics act upon our senses to make us feel more, hear more and see more than we otherwise might… feelings are intertwined with mental, physical, spiritual and social health'.[18 p4] Several studies explored the relationship between arts, health and wellbeing and a growing body of evidence suggests that cultural participation (including music, art making, theatre, dance, museum and heritage activities) enhances human health and wellbeing.[1, 19-27] Research conducted in museums-and-health, for example, suggests that 'museum and art gallery encounters can help with a range of health issues, enhance wellbeing, and build social capital and resilience'.[27 p286] According to research conducted in Norway with over 50,000 adults,[28] participation in both receptive/passive and creative/active cultural activities was significantly associated with good health, life satisfaction, and low anxiety and depression. Findings demonstrated how manual creative practices such as knitting had a positive impact on people suffering from depression and post-traumatic stress disorder because 'the movements involved in knitting are bilateral, rhythmic, repetitive, and automatic'.[28 p40] The authors attributed the positive mood of knitters to enhanced production of serotonin resulting from repeated movements, and that bilateral processes appeared to engage brain capacity and facilitate a meditative-like state more readily than unilateral ones. Although creative activities are seen to provide major benefits for refugees and asylum seekers, researchers have become 'increasingly conscious of the value of recording and analysing what has been happening'.[8 p1]

The current study was conducted in the light of mental health and arts-in-health/museums-in-health evidence, and the large number of organisations that engage migrants, refugees and asylum seekers in the arts, with nearly 200 of these in the UK.[29] The current research was conducted at the Helen Bamber Foundation (HBF), a charity offering support to refugees and asylum seekers as victims of torture and human rights violations. HBF adopts a holistic approach to support its clients that includes cultural activities within a three-phase model of integrated care comprising stabilisation (medical and legal support), intervention (trauma-

For peer review only - http://bmjopen.bmj.com/site/about/guidelines.xhtml

focussed therapy and general psychological care), and integration (attending the creative, computing and English classes, and interacting with other clients). The model is based on the needs of refugee/asylum seekers who often require assistance on several fronts (e.g. psychological support might not be effective if a person does not have a place to sleep or lives in a dangerous situation, though giving people a place to sleep is not enough to overcome trauma). Their creative arts programme (CAP) led by volunteers includes art (painting and drawing), photography, textiles (dressmaking and knitting) and singing, and is attended by c.100 clients. The study took the view that arts participation would enhance the sense of wellbeing by allowing participants to form meaningful relationships based on trust and mutual support (improving social wellbeing) and enhance self-esteem and self-confidence (increasing personal wellbeing).

To provide voice and agency for participants, the study used a collaborative approach to explore client experiences of creative activities with HBF clients, staff and volunteers acting as co-researchers. The research was operationalised according to the principles of participatory action research (PAR)[30, 31] as a 'way of opening up space for dialogue and conversation',[32 p28] aiming to 'understand and also improve a particular situation',[32 p1] both pertinent aspects when working with displaced people. The PAR approach encourages an active contribution in the production of knowledge within a collaborative framework, emphasising 'equal partnerships',[33 p167] and the 'role of the participant in the design, implementation, and dissemination of the research'.[34 p78] The collaborative character of PAR is grounded in efforts to 'democratise the research process',[35 p3] so 'equal weight and consideration is given to the contributions of both the community and academic partners'[36 p170] again valuable for everyone involved but specifically refugees/asylum seekers. One of the pillars of PAR, especially when community-based is ownership.[37]

By offering equal weight to client contributions focusing on the relationship between forced displacement and arts participation, the study aimed to empower participants by developing their sense of ownership in the project. Through collaborative working between the charity, displaced people and academics, the purpose of the research was to co-develop a methodological approach to address the needs of refugees/asylum seekers and to co-produce interview questions that could be applied in UK and international settings. The objectives were to expand on arts-in-health and collaborative research approaches by exploring the benefits of engagement in cultural and creative activities on the health and wellbeing of forcibly displaced people and contribute to the current debate on migration and public health.

For peer review only - http://bmjopen.bmj.com/site/about/guidelines.xhtml

Method

Design

Qualitative research conducted comprised observation, focus groups and one-to-one semi-structured interviews with clients, volunteers and staff as co-researchers within a collaborative PAR approach.[38]

Participants

The study recruited an opportunity sample of volunteer participants through information leaflets and contact details in reception. Clients were approached by the researcher using two recruitment criteria: i) clients had received therapy for at least two years prior to the research and had entered the integration stage of the model of integrated care; this meant that though still vulnerable, clients had established a therapeutic relationship of trust enabling them to form safe relationships in the wider community and move forward with their lives; and ii) they had attended at least one of four CAP groups (art, photography, singing and textiles) for around two years. All participants spoke English sufficiently well to participate having attended English classes for about two years as part of integrated care. In total, 31(6m) participants volunteered for the study and attended focus group comprising 12(2m) refugees/asylum seekers, 4(1m) volunteers, and 15(3m) charity staff. Of these, 17(3m) participated in interviews comprising 7(1m) refugees/asylum seekers, 7(1m) volunteers, and 3(1m) staff.

Patient and public involvement

HBF staff were instrumental in the application for research funding and collaborated with the researcher to determine the study's objectives. HBF clients were involved in the recruitment process by recommending that other clients join them in the research, though the researcher ensured that they matched recruitment criteria (above). In keeping with PAR, research questions and outcome measures concerning the effects of cultural and creative activities on the psychosocial wellbeing of refugees/asylum seekers were determined by co-researchers (HFB clients, volunteers and staff) with differing involvement depending on their role at the charity. Focus groups and interviews gave voice to clients' priorities and preferences but, due to their vulnerability, no personal information was requested as the process of remembering could have been difficult.[7, 10] Although some participants mentioned personal issues and experiences, they were not encouraged to do so. Data generated by co-researcher involvement

For peer review only - http://bmjopen.bmj.com/site/about/guidelines.xhtml

in the research collaboration informed focus groups, in turn focus group outcomes led to the development of questions for the in-depth interviews to follow. In addition to participation in focus groups and interviews, a core group of seven co-researchers (3 clients, 2 volunteers, 2 staff, with 1 male per category) were involved in design of the interview guides, discussion of emerging themes and dissemination of the research findings to other participants for verification and comments, in accordance with PAR principles.[33] The results of the research were fed back to everyone involved with HBF through on-going communication with the researcher, an internal report lodged at the charity, and an end-of-project event.

Data collection

Data collection carried out by the location-based researcher consisted of three stages: Stage 1 (months 1-2) participant observation; Stage 2 (months 3-4) focus groups; and Stage 3 (month 5) semi-structured interviews. In Stage 1, participant observation was used as a starting point 'for studying how organisations work, the roles played by different staff and the interaction between staff and clients'.[38 p32]. The researcher attended creative classes for clients and spent a day a week working from HBF (12-15 hours per week) to determine the nature of focus groups. In Stage 2, four focus groups were held, first and final with clients (n=8, n=4 respectively), second with volunteers (n=4), and third with staff (n=15). In the focus groups, participants were asked to discuss research questions (Table 1) involving ways of gathering information on the impact of creative activities, and reasons for attending groups (or in the case of volunteers and staff, reasons for involvement). All focus groups were organised informally to facilitate development of discussion among participants and allow an exchange of experiences and ideas. Ideas that emerged during focus groups laid the basis for first drafts of interview questions, tailored to clients, volunteers or staff (Table 2). In line with the collaborative ethos, drafts were circulated to receive feedback from co-researchers prior to the interview stage, and volunteers and staff were consulted and asked to provide comments and suggestions for interview questions. For Stage 3, clients (n=7), volunteers (n=7) and staff (n=3) participated in interviews with the researcher using a semi-structured format to allow free expression within the research constraints and limit potential research bias.[39, 40]

INSERT TABLE 1 ABOUT HERE

INSERT TABLE 2 ABOUT HERE

For peer review only - http://bmjopen.bmj.com/site/about/guidelines.xhtml

Data analysis

A first informal thematic analysis was conducted by the researcher while writing field notes consisting of a chronicle of descriptive rather than analytic events both observed and provided by co-researchers as the 'raw material of the research'.[30 p38] The process of transcribing notes was used to identify key points connected to the research question. This preliminary analysis informed focus group topics where points were discussed more in detail. After the first round of focus groups, all notes were compiled into a Word document and repeatedly read by the researcher and co-researchers to search for recurring topics and themes. Thematic analysis of focus group outcomes were explored in detail during interviews. All interviews were recorded and transcribed to become familiar with the data and begin the coding process [37]. All details in the transcripts were recorded verbatim (e.g. sighs, laughter, silences, and tears) as their exclusion could have changed the meaning expressed.[30] Transcriptions were uploaded into NVivo11 to produce nodes from coding-relevant concepts. NVivo's word frequency query facilitated the search for key words (including stem words and synonyms) grouped under the same theme. The text search query provided a comprehensive analysis of data. After coding of data into initial themes,[37] a review of themes followed to refine the analysis.

Results

Focus Groups

Focus group data were analysed using thematic analysis (NVivo11). Findings showed consistency in the way all HBF participants (clients, volunteers and staff) articulated their reflections on the impact of creative activities. The benefits of creative activities were highlighted in clusters emerging from around three main overarching themes: 'skills', 'social aspects/friendships' and 'mood-personal sphere'; the latter sub-divided into 'brain' (creative thought processes), 'routine', self-expression' and 'confidence'. During focus groups, co-researchers were asked to write answers to the questions posed, and a collective discussion followed. Clients responded consistently about benefits of the creative programmes. One client listed 'Activities help my brain think about other stuff and keep me busy and relaxed. You can't think about bad things. To learn more skills. I can use skills to help others.'

Learning new or improving existing skills was a key theme emerging out of focus groups, as skill development appeared to change clients' perceptions of their status; as a female volunteer at HBF for two years suggested 'participants do not feel assessed as clients, they are just learners or artists. These groups paved the way for identifications different from

For peer review only - http://bmjopen.bmj.com/site/about/guidelines.xhtml

being a victim, a refugee or an asylum seeker'. Clients also mentioned how acquiring new skills was an important factor in improving self-esteem; one client wrote about attending CAP 'Give me confidence. Make me feel good and useful. Change my mood. Learn new things and meet people. Improve my skills. Give me hope to lead a better life'.

Learning new skills was a benefit connected to the second theme that emerged during focus groups, the social aspect. it allowed people to meet others in similar situations and create friendships and/or a support network; as a client wrote 'Allows me to feel included. Safe environment. Improves my mood. Learn new skills. Gets me out of the house. Meet new people'. Staff too recognised the social aspect of activities as a major benefit, with one suggesting 'in these groups, clients learn to have balanced, reciprocal relationships, which they have not experienced before or for a long time given their experience of violence (trafficking). They provide clients with an alternative identity, i.e. they are not simply victims here, they are learners, artists, dressmakers, etc.' Similar themes emerged in the volunteer focus group during which a relatively new male volunteer involved of four months was impressed by the solidarity between clients, emphasising the benefits of the 'feeling of belonging to a group' and 'strong support that people receive from other members'.

The third and final theme that emerged when discussing the benefits of CAP the mood-personal sphere. Staff commented on the mood enhancing benefits of CAP for people with PTSD; one staff member observed 'creative activities allow clients to momentarily leave out their problems or memories, by focusing on a particular activity they cannot think about anything else'. The focus group with staff emphasised that being busy in the company of others allowed clients to feel safe and not to dwell upon their own situation. In concentrating on learning new skills, clients blocked out (albeit for short periods) memories of the past. Importantly, responses illustrated that focus group participants perceived their involvement as beneficial at multiple and overlapping levels. Enhancement of mood appeared to derive from not only meeting new people, forging meaningful relationships and feeling part of a group but also connected to a growing self-confidence and self-esteem experienced as clients acquired new skills.

Interviews

During interviews, clients elaborated on practical and social skills, and commented on the positive effect of attendance on their mood and emotion:

For peer review only - http://bmjopen.bmj.com/site/about/guidelines.xhtml

Practical skills: Learning new practical skills or improving those previously learnt emerged strongly among asylum seekers without the right to work. As a client pointed out:

I have learned a lot of skills: I've learned sewing, now I'm learning art, I can do better, before I didn't know how to draw a person, to mix colours, you know such things... so you keep on learning, slowly by slowly, then in the end you find yourself, you're a professional, so that is very great... to me... I like to come, always and attend, and listen to what they tell me, and I do it.

Or, as another client explained:

I've learned new skills [...] and you can never waste your time learning new skills. New skills always help you in your life, always. So, everything I've learned will help me. You think like 'film club, learning how to edit, etc.'. People pay people on YouTube to edit their films, and I'm, like, I could just do my own, if I wanted to have a YouTube channel, I can do my own.

Skill improvement provided a sense of achievement and improved self-confidence, talking about learning new skills, one client observed:

When you are in this situation it feels like life has, in a way, stopped. And you can't do anything to change it. But by doing all this activity you feel, or I felt…it was like, you know, that I was learning something in my life, rather than just waiting.[…] For instance, if I go for a job somewhere, where you have to write what skills you have, so I could include all of this. I mean I don't have a certificate or, like, proper qualifications, but I've learned all those skills, so I feel like, it is, in a way, impressive? Because you feel like, you know, rather than just waiting and not doing anything, you have been learning.

The sense of self-improvement associated with learning skills was linked to the perception that by learning, clients were preparing themselves for a new life when they would be allowed to live and work in the UK, countering the perception that life for them had stopped and giving them something to look forward to. The process of skills learning was not simply a vertical one; peer learning was equally important, stressing the importance of social aspects.

Social skills: The opportunity to overcome social isolation and create a social network emerged as one of the benefits of creative engagement. As one client indicated:

The groups changed me because you know when I come, I feel lonely and doesn't have anybody. I was lonely I don't even know whether there will be a home from morning to live in. But when I started coming these groups, I told you I feel active, I feel coming to

For peer review only - http://bmjopen.bmj.com/site/about/guidelines.xhtml

see people, that I speak to. I don't even know how to communicate with people before. I told you because of my coming here I now feel to communicate to people. Before I never communicate to people, I don't know how to do it. And I don't know how to play with people. But these groups make me now taught me: play with people, to meet me.

The client expressed the difficulties of establishing new relationships and the feeling that the group was a place in which they could regain this ability to connect with other people. The social aspect was an important point reiterated in all interviews; fellow clients were described as 'friends' but also as 'brothers and sisters'. Arts activities allowed clients to create a community characterised by solidarity, as one client explained:

If you're in a situation where you've been completely isolated from people for a while and you just don't know who to trust, or to be around people, it's one of those spaces where you can get to meet people, socialise, and actually make friends.[…] And these friendships do last. A life-time, because you understand what the person has gone through, you don't have to explain a lot, you know, they just know that you've been through something terrible.[…] So, it's easier when you have people here who just understand what you've possibly been through.

This remark connects with the observation that 'engagement in participative creative arts activities in communities can help to build social capital, address loneliness and social isolation, and build personal confidence and a sense of empowerment'.[25 p32] The awareness of experiencing similar situations makes clients feel free to share their own experiences with one another but also not to, if they did not feel like opening up:

There is a sense of community: you know you go there, you know there are people like you in the same situation you're not going to be judged, so it's that sense of community: we know what's going on, we don't have to talk about it.[...] It's a distraction from immigration and we know we are all going through it, but we don't have to talk about it. There are other things going on in life, and we talked about, for example in the art group, the works we produced and what we could do, what we could achieve and get inspired by each other's work.

Again, this remark introduced the third broad theme which emerged out of the interview analysis, which was the impact of creative arts on personal mood and emotion.

Mood and Emotion: Learning or improving skills while forging long-lasting relationships in a context of deep social isolation (outside of HBF), inevitably had a substantial impact on mood and emotion. In discussing this aspect of creative arts, interviewees focused

For peer review only - http://bmjopen.bmj.com/site/about/guidelines.xhtml

particularly on how groups allowed them to have a routine, a 'luxury' one client said 'when you can't do nothing but waiting for the government to decide on your right to stay in the country'. Routine combined with skills learning/improving was thought to have a positive impact on clients' mental health; 'having something to do' and 'something to look forward to' were recurrent expressions during interviews. As one client noted:

I used to come here every day of my life: Monday, Tuesday, Wednesday... because I don't have anywhere to go, so I just come here, and I attached myself to the groups, we met, we talk, we... then I've gone to knit, I've gone to do computer, reading, many things I learnt here, so I just like it.

Another client elaborated further:

It's something that I look forward to. One thing you have to bear in mind is that I am not working, and I am not studying, you know, so it was the only thing that I would look forward to because it was something to do, otherwise I would just be at home, doing nothing, you know, feeling very sorry for myself, getting upset all the time. I mean I still feel that way but at least there's something to look forward to, when you don't have hope or something to look forward to, it increases your depression levels, so for me it's helped me greatly.

This statement describes the social and emotional isolation as part of post-migration living difficulties. The routine of classes, together with an awareness that others in similar situations would attend, provided comfort to clients, countering feelings of isolation:

There is nothing else for you and it's also a way of, like, using your time wisely, 'cause at the end of the day most of the time you're just sitting down doing nothing, and doing nothing slowly begins to affect your mind, your brain, you know you become lazy, you know you literally are just in a four-squared room[...]

The benefits of a routine were increased by inclusion of creative activities that according to clients and volunteers, had a positive impact on mood because they exercised the brain and allowed clients to find a new language to express themselves. As a client stated:

[Attending creative groups] gives you a lot of confidence, you know. And it allows your brain to think outside of the box because I think we are just in that box and... but when you attend these classes, your mind, you know, opened and you learn new things and you want to then learn other things as well.

This comment highlights an important link between arts and feelings; as another stated:

It is more of expressing, more of letting go, it's like getting a spirit out of you, and you don't have necessarily have to tell someone 'I've done this because of that and that, and

For peer review only - http://bmjopen.bmj.com/site/about/guidelines.xhtml

this', you know. Because sometimes you just don't find the voice to talk about it and, I, as a person am really shy and, you know, I feel easily embarrassed, you know.[...] So... that's why I'm into arts, yes I'm doing arts really.

Clients agreed that the possibility of self-expressing without having to articulate their feelings, helped them grow emotionally and gain confidence. It was not only clients who benefited from CAP; findings also pointed to the positive impact on volunteers, a point stressed by all volunteers when discussing personal experiences.

Discussion

Creative and cultural activities were observed over a sustained period of five months. Clients participating in the activities had reached the integration stage of the model of integrated care consequently had been taking part in CAP activities for two plus years prior to the research. Activities provided refugees and asylum seekers with new skills, including practical and technical skills, and social and life skills involving language acquisition obtained partly through informal peer learning and mutual support. Learning new skills contributed to the sense of wellbeing and empowerment experienced by HBF clients. The time spent by the researcher at the HBF was vital for familiarisation with the context, getting to know people involved in the organisation, and explaining the research to recruit participants from among clients, volunteers and staff. The fact that several clients attended more than one group per week (2-4) made it possible to forge trusting relationships and recruit co-researchers, allowing them to feel comfortable in speaking their mind. A possible issue here was that the number of groups attended by clients was not accounted for, so it was not evident as to whether increased participation furthered benefits at a higher/faster level. Once recruited, participants remained with the study in their co-researcher roles and continued to attend activities on a weekly basis for the five months (except for rare absences due to illness).

Activities appeared to positively enhance mood and emotion both for clients and volunteers facilitating CAP groups. Although passive participation was not compared,[28] active and creative participation in the groups specifically benefited the clients. A key reason was that asylum seeker status in the UK would not have permitted alternative occupation or employment, so clients might not have otherwise left their homes or met people on a regular basis. In keeping with previous research, participants reported that participation in cultural and social activities contributed to their social health,[18] and aligned with a growing body of evidence[1] to suggest that creative activities enhanced mental wellbeing,[19-27] helping clients to develop self-confidence and resilience.[21] Client preferences for activities

For peer review only - http://bmjopen.bmj.com/site/about/guidelines.xhtml

indicated that singing was their favourite group, followed by photography, art and textiles. Singing was regarded as beneficial because it allowed people to meet socially and work collaboratively, aspects considered important in countering loneliness. Findings aligned with a recent qualitative study indicating that choral singing promoted improvements in social, emotional, physical, and cognitive functioning and that benefits were experienced similarly irrespective of age, gender, nationality.[41] In the current study, photography, art and textiles were praised by clients for the skills taught and although social aspects were relevant, skills that allowed participants to continue activities at home (e.g. sewing, drawing) were highly valued. Interviewed staff were asked to comment on the popularity of activities, and although the order was the same as that of clients, they were not asked their opinion on which were most beneficial.

The current study bridged the gap between two research disciplines: arts-in-health and forced displacement and mental health. It contributed to existing literature by demonstrating beneficial effects of creative activities on the wellbeing of refugees/asylum seekers and showed that PAR was an appropriate and 'democratic' means of collaboration between displaced communities and academics.[40 p3] In contrast with traditional research where 'academics benefit from the research, but often the people involved hear very little if anything from the researcher again',[33 p78] a core element of PAR is that research should be meaningful and have tangible outcomes for co-researchers as they work together to bring about significant change within their community or society at large. In the current study, client involvement in the research and their decision to organise a public exhibition to showcase their artwork was a means for them to interact with wider society and actively re-write the narrative around asylum seekers and refugees, often depicted in negative terms in the public sphere.[42]

Adopting a PAR approach is important when working with immigrant communities as it ensures that the research question is of 'genuine importance',[34 p118] and facilitates translation of research findings into 'actions that will benefit the community'.[34 p119] PAR was particularly useful when conducting research with refugees/asylum seekers with PTSD and other mental health issues because the process of involvement as co-researchers countered client isolation and built social capital through regular meetings. In helping to interpret findings, clients acquired research methods skills (e.g. drafting interview guides, conducting focus groups, discussing data) and organisational abilities (event/exhibition). Participating in the research and disseminating findings gave clients a sense of agency and ownership of the project that enhanced self-confidence and feelings of empowerment, one of

For peer review only - http://bmjopen.bmj.com/site/about/guidelines.xhtml

the pillars of PAR especially when community based.[35 p2] Participation provided a positive means of expression countering their refugee/asylum seeker status that otherwise, rendered them effectively powerless.

In terms of study weaknesses, the greater number of female than male participants was probably due to the fact that most creative groups were led by female volunteers (2m, 8f). Most clients attending these groups were also female except for singing, where numbers were approximately equal (although only one male choir member took part in a focus group). Given the nature of the singing activity, conversation was limited to the first minutes before arrival; once rehearsals had started it was difficult for the researcher to form relationships with clients. It was also possible that some activities were seen by clients as gender-neutral (e.g. photography or singing), but others such as working with fabrics and soft materials (e.g. wool and cloth) may have been seen as a predominantly female activity; male participants might have been more comfortable working with harder materials (e.g. wood and metal). It was generally more difficult to engage male participants in the research; a possible reason for this was female clients might have found it easier to relate to the female researcher. A further weakness was low attendance for focus groups possibly due to difficulties of organising them at convenient times for everyone, given changing client circumstances (e.g. interviews for benefits, housing etc.). More clients expressed an interest than those who participated, nevertheless were happy to informally discuss topics debated. Others wanted to be kept in the loop without participating in focus groups and interviews, so research methods were discussed informally during meetings between researcher and clients.

Conclusions

This study expanded on arts-in-health and collaborative research approaches by exploring the effects of cultural and creative activities on the health and wellbeing of refugees and asylum seekers. Creative activities at HBF provided opportunities for clients to socialise and develop new relationships based on respect and mutual recognition. In turn, these opportunities countered loneliness and allowed refugees/asylum seekers to develop a sense of belonging in a safe space. The opportunities further supported freedom of expression and the possibility of developing a new identity to counter the negative stereotype of being viewed as a refugee or asylum seeker,[42] leading to sustained improvements in positive mood and self-confidence, and countering anxiety, depression and other mental health issues. By focusing on the relationship between arts, wellbeing and forced displacement and in keeping with PAR, the study actively changed the narrative surrounding refugees/asylum seekers and gave an

For peer review only - http://bmjopen.bmj.com/site/about/guidelines.xhtml

For peer review only

understanding of how to improve conditions for forcibly displaced people through opportunities for social connectivity, creative thinking and employment skills. It is recommended that other refugee/asylum seeker organisations could utilise the interview questions generated by the study or develop their own, following PAR guidelines, to appraise and improve migrant provision particularly in terms of up-skilling peer-learning. If scaled up across the UK, programmes of creative activities for migrants could reduce pressure on health and social care services and help to address the social determinants of health inequality.[16] Research outputs have wider implications for policy development, for example by the United Nations Refugee Agency, World Health Organisation, and other government and non-government agencies.

Contributors

CC, LT and HC made substantial contributions to the conception and design of the work; drafting and critically revising the work for intellectual content; and the final approval of the version published. CC conducted the acquisition, analysis and interpretation of data for the work. All authors agree to be accountable for all aspects of the work. We would like to thank the clients, volunteers and staff who were co-researchers in the project.

Funding

The work was supported by the Global Challenges Research Fund (GCRF) Grant reference: ES/P003818/1 PI: HC

Ethics

The study gained approval from UCL Ethics Committee (Ethics Application 4526/002 Co-developing a method for assessing the psychosocial impact of cultural interventions with displaced people: Towards an integrated care framework) to carry out the research with potentially vulnerable participants.

Competing interests

The authors have no competing interests.

Data sharing statement

Due to the confidential nature of the human participant data, it will not be freely available via free access databases. Any request for data should be addressed to the corresponding author.

For peer review only - http://bmjopen.bmj.com/site/about/guidelines.xhtml

References

1. All-Party Parliamentary Group on Arts, Health and Wellbeing (APPGAHW). Creative Health: The Arts for Health and Wellbeing. Inquiry Report. 2017; http://www.artshealthandwellbeing.org.uk/appg-inquiry/
2. New Economics Foundation (NEF). 2009. National accounts of wellbeing: What is wellbeing? NEF. http://www.nationalaccountsofwellbeing.org/learn/what-is-well-being.html
3. New Economics Foundation (NEF). National Accounts of Well-being: Bringing Real Wealth onto the Balance Sheet. 2009. https://neweconomics.org/uploads/files/2027fb05fed1554aea_uim6vd4c5.pdf
4. Ander E, Thomson LJ, Noble G, Lanceley A, Menon U, Chatterjee H. Generic well-being outcomes: Towards a conceptual framework for well-being outcomes in museums. Int J Museum Manage Curator 2011; 26:237–259. doi:10.1080/09647775.2011.585798
5. Crawley H, Düvell F, Jones K, McMahon s, Sigona, N. Destination Europe? Understanding the dynamics and drivers of Mediterranean migration in 2015. MEDMIG Final Report 2016. www.medmig.info/research-brief-destination-europe.pdf
6. Vaughan-Williams N. "We are *not* animals!" Humanitarian border security and zoopolitical spaces in Europe. Political Geography 2015;45:1–10. doi:10.1016/j.polgeo.2014.09.009
7. Katona C. Non-affective psychosis in refugees. Br Med J 2016;352:i1279. doi:10.1136/bmj.i1279
8. Robjant K, Robbins I, Senior V. Psychological distress amongst immigration detainees: A cross-sectional questionnaire study. Br J Clin Psychol 2009;48:275–286. doi:10.1348/014466508X397007
9. Alpak G, Unal A, Bulbul F, Sagaltici E, Bez Y, Altindag A et al. Post-traumatic stress disorder among Syrian refugees in Turkey: A cross-sectional study. Int J Psychol Clinic Practice 2015;19:45–50. doi:10.3109/13651501.2014.961930
10. Schubert CC, Punamäki R. Mental health among torture survivors: cultural background, refugee status and gender. Nordic J Psychol 2011;65:175–182. doi:10.3109/08039488.2010.514943
11. Silove D, Sinnerbrink I, Field A, Manicavasagar V, Steel Z. Anxiety, depression and PTSD in asylum-seekers: associations with pre-migration trauma and post-migration stressors. Brit J Psych 1997;170:351–357. doi:10.1192/bjp.170.4.351

For peer review only - http://bmjopen.bmj.com/site/about/guidelines.xhtml

doi:10.1348/014466508X397007

12. Aragona M, Pucci D, Mazzetti M, Geraci S. Post-migration living difficulties as a significant risk factor for PTSD in immigrants: a primary care study. Italian J Public Health 2012;9:e7525;1–8. doi:10.2427/7525
13. Allen J, Allen M. The social determinants of health, empowerment and participation. In S Clift, P Camic (Eds.), Oxford Textbook of Creative Arts, Health, and Wellbeing: International Perspectives on Practice, Policy and Research. New York, Oxford: Oxford University Press; 2016, p27–34.
14. Fisher M. Why mental health is a political issue. The Guardian, July 16, 2012. https://www.theguardian.com/commentisfree/2012/jul/16/mental-health-political-issue
15. Griffin J. The Lonely Society. London: The Mental Health Foundation. https://www.mentalhealth.org.uk/sites/default/files/the_lonely_society_report.pdf
16. Marmot M, Allen J, Goldblatt P, Boyce T, McNeish D, Grady M et al. Fair Society, Healthy Lives: The Marmot Review. 2010; http://www.instituteofhealthequity.org/resources-reports/fair-society-healthy-lives-the-marmot-review/fair-society-healthy-lives-full-report-pdf
17. Paul K, Moser K. Unemployment impairs mental health: Meta-analyses. J Vocational Behavior 2009;74:264-282. doi:10.1016/j.jvb.2009.01.001
18. Prior RW. Editorial. J Applied Arts Health 2009;1:3–6.
19. Bygren LO, Johansson S, Konlaan BB, Grjibovski AM, Wilkinson AV, Sjöström M. Attending cultural events and cancer mortality: A Swedish cohort study. Arts Health: Int J Res Pol Pract 2009;1:64–73. doi:10.1080/17533010802528058
20. Camic P, Chatterjee H. Museums and art galleries as partners for public health interventions. Perspect Public Health 2013;133:66–77.
21. Chatterjee H. Museums and art galleries as settings for public health interventions. In S Clift, P Camic (Eds.), Oxford Textbook of Creative Arts, Health, and Wellbeing: International Perspectives on Practice, Policy and Research. New York, Oxford: Oxford University Press 2016; p.281–289.
22. Clift S, Skingley A, Coulton S, Rodriguez J. The effectiveness and cost-effectiveness of a participative community singing programme as a health promotion initiative for older people: protocol for a randomised controlled trial. BMC Public Health 2011;11:1–6. doi:10.1186/1471-2458-11-142

For peer review only

23. Clift S, Camic P. Oxford Textbook of Creative Arts, Health, and Wellbeing: International Perspectives on Practice, Policy and Research. New York, Oxford: Oxford University Press, 2016.

24. Konlaan BB, Bygren LO, Johansson S. Visiting the cinema, concerts, museums or art exhibitions as determinant of survival: a Swedish fourteen-year cohort follow-up. Scand J Public Health 2000;28:174–178. doi:10.1177/14034948000280030501

25. Staricoff RL. Arts in health: A review of the medical literature. London: Arts Council England, Research Report 2004;36:1–91. http://www.artsandhealth.ie/wp-content/uploads/2011/08/AHReview-of-Medical-Literature1.pdf

26. Staricoff RL. Arts in health: The value of evaluation. Perspect Public Health 2006;126:116–120. http://journals.sagepub.com/doi/pdf/10.1177/1466424006064300

27. Chatterjee HJ, Noble G. Museums, Health and Wellbeing. Farnham: Ashgate, 2013.

28. Cuypers K, Krokstad S, Holmen TL, Knudtsen MS, Bygren LO, Holmen J. Patterns of receptive and creative cultural activities and their association with perceived health, anxiety, depression and satisfaction with life among adults: the HUNT study, Norway. J Epidemiol Community Health 2012;66:698–703. https://jech.bmj.com/content/jech/66/8/698.full.pdf

29. Kidd B, Zahir S, Khan S. Arts and Refugees: History, Impact and Future. London: Arts Council England, 2008. https://baringfoundation.org.uk/wp-content/uploads/2014/10/ArtsandRefugees.pdf

30. Kemmis S, McTaggart R, Nixon R. The Action Research Planner: Doing Critical Participatory Action Research. Singapore: Springer 2014.

31. Bradbury H. Introduction: How to situate and define action research. In H Bradbury (Ed.). The Sage Handbook of Action Research, London: Sage 2015. p.1–9.

32. Nicolaidis C, Raymaker DM. Community based participatory research with communities defined by race, ethnicity, and disability: Translating theory to practice. In H Bradbury (Ed.), The Sage Handbook of Action Research. London: Sage 2015. p.167–178.

33. Daykin N, Stickley T. The role of qualitative research in arts and health. In S Clift, P Camic (Eds.), Oxford Textbook of Creative Arts, Health, and Wellbeing: International Perspectives on Practice, Policy and Research. New York, Oxford: Oxford University Press 2016, p.73–82.

34. Vaughn LM, Jacquez F. Community-Based Participatory Research Studies Involving Immigrants. In S Coughlin, SA Smith, ME Fernandez (Eds.), Handbook of Community-

For peer review only

Based Participatory Research. New York, Oxford: Oxford University Press, 2017. p.115–130.

35. Blumenthal DS. Is Community-based participatory research possible? Am J Prev Med. 2011;40:386–389.
36. Braun V, Clarke V. Using thematic analysis in psychology. Qual Res Psychol 2006;3:77–101.
37. Pope C, Mays N. Qualitative Research in Health Care, 3rd Edition. Oxford: Blackwell, 2006.
38. Pope C, Ziebland S, Mays N. Analysing Qualitative Data. In C Pope, N Mays. Qualitative Research in Health Care, 3rd Edition. Oxford: Blackwell. 2006. p.63–81.
39. Kemmis S, McTaggart R, Nixon R. The Action Research Planner: Doing Critical Participatory Action Research. Singapore: Springer 2014.
40. Bradbury H. Introduction: How to Situate and Define Action Research. In H Bradbury (Ed). The Sage Handbook of Action Research, London: Sage 2015. p.1–9.
41. Livesey L. Morrison I. Clift S. Camic P. Benefits of choral singing for social and mental wellbeing: Qualitative findings from a cross-national survey of choir members. J Pub Mental Health 2012;11:10–26.
42. Khosravi S. 'Illegal' Traveller: An Auto-ethnography of Borders. Basingstoke: Palgrave MacMillan 2010.

For peer review only - http://bmjopen.bmj.com/site/about/guidelines.xhtml

Table 1. Focus group questions

Clients:
1. Why do you like the HBF creative arts programme?
2. How would you gather information or evidence about the benefits of artistic activities?
3. Which creative arts classes have a positive impact on the lives of refugees? Circle the option(s) that you think work best or add another one. Arts and crafts Drawing Films Knitting Photography Singing Textiles.
Volunteers:
1. Why do you volunteer at the HBF?
2. How do you think your group benefits clients?
3. How would you collect evidence on the effects of participating in artistic activities?
Staff:
1. Why do you recommend clients to attend Creative Arts groups?
2. What do you think are the benefits of attending arts groups?
3. How would you collect evidence on the effects of participating in artistic activities?

For peer review only

1 Table 2. Interview questions

Clients:

1. How long have you been attending the CAP classes for?
2. Which groups do you attend?
3. Why did you choose this/these groups(s)?
4. What do you like the most about these groups?
5. Is there anything about the groups that you find difficult?
6. Anything you would like to change?
7. Is there a group you prefer? Which one? Why?
8. How do you feel when you attend this group?
9. Do you think there are benefits of attending these groups? If so what are they?
10. During our previous meeting, it emerged that participants feel that groups allow them to learn new or improve already acquired skills. Do you agree? Have you learnt any new skill? And what do you think about the possibility of learning new skills? (How does that impact your life)
11. Another element which emerged during our meeting is that people enjoy the social aspect of these groups: meeting people and finding new friends. What do you think about this? [supporting questions: do you like being around other people? Why? What happens when you attend a session? Have you made new friends? How do you feel about that?]
12. Among all of the reasons why you enjoy attending the creative arts group, which one is the one that you feel strongest about?
13. Why do you keep attending?
14. If you could compare the way you were feeling before joining any of these activities and the way you feel now, would you say you feel any different? Explain
15. Creative activities of course are part of the support that HB offers to its clients. Do you think that you would feel the same about yourself today even without attending these creative activities groups? Why?
16. Do you think that taking part in these creative activities has had any influence on the way you see yourself? And the way you see yourself in London?
17. If you were to recommend someone to join a group, what would you say?

For peer review only - http://bmjopen.bmj.com/site/about/guidelines.xhtml

Volunteers:

1. What do you teach?
2. How long have you been volunteering for?
3. Why did you decide to volunteer?
4. How did you learn about the Helen Bamber Foundation?
5. What do you think your group offers clients?
6. What are the main challenges you face/have faced as a volunteer?
7. Do you find it difficult to engage clients in the activity you coordinate? What do you think clients like about the way you (and your colleagues) handle the sessions? Is there anything that clients don't like?
8. How would you describe a typical session?
9. What do you think people like the most about your group?
10. Do you ever discuss emotions with clients? Or their personal situation?
11. Look at the notion of wellbeing defined by NEF (New Economics Foundation):

Wellbeing as 'the dynamic process that gives people a sense of how their lives are going through the interaction between their circumstances, activities and psychological resources or 'mental capital.' (NEF 2008 p3).

According to the NEF, there are different components to wellbeing, such as: *'Personal wellbeing (emotional wellbeing, satisfying life, vitality, resilience, self-esteem and positive functioning) and social wellbeing, including supporting relationships and trust and belonging.'* (NEF 2009).

12. Do you think that the activity you lead has any impact on the wellbeing of clients? Elaborate
13. If you were asked to recruit people for your group, what would you say to convince them to join?
14. Would you use different words to promote your group with men and women? Why?
15. Do you think that your group makes a difference in the lives of clients? How? Why?
16. And what about your own life? What is the impact of your volunteer activity on your own life?

For peer review only - http://bmjopen.bmj.com/site/about/guidelines.xhtml

Staff:

1. How long have you been working at HB for?
2. Why did you decide to work here?
3. What is your role?
4. As part you work, do you have any direct contact with clients?
5. Could you say a few words on your relationship with clients?
6. How would you describe your own experience at HB so far?
7. What are the main challenges that you face/have faced in your position?
8. Do you think that your own gender influences the ways in which clients relate to you?
9. In your position, do you discuss clients' personal situations and feelings?
10. If so, do you think that the cultural background of clients affects the way in which you discuss emotions? If it does, how and why?
11. What are the main challenges of discussing emotions with clients?
12. Could you say a few words on the Model of Integrated Care? Do you think it works? And if so, why?
13. Do you recommend clients to attend any of the CAP groups? What do you say in this case?
14. What role do you think cultural and creative activities play in the recovery of clients?
15. Are there any particular activities that, in your opinion, are more popular with clients?
16. Would you say that the programme makes a difference in the lives of clients? If yes, please elaborate.
17. Have you witnessed clients benefiting from their own participation in the creative arts programmes? If yes, in what ways?

For peer review only

Research and reporting methodology
Revised **Standards for Quality Improvement Reporting Excellence** (**SQUIRE 2.0**) publication guidelines

Notes to authors

- The SQUIRE guidelines provide a framework for reporting new knowledge about how to improve healthcare.
- The SQUIRE guidelines are intended for reports that describe system level work to improve the quality, safety and value of healthcare, and used methods to establish that observed outcomes were due to the intervention(s).
- A range of approaches exists for improving healthcare. SQUIRE may be adapted for reporting any of these.
- Authors should consider every SQUIRE item, but it may be inappropriate or unnecessary to include every SQUIRE element in a particular manuscript.
- The SQUIRE glossary contains definitions of many of the key words in SQUIRE.
- The explanation and elaboration document provides specific examples of well-written SQUIRE items and an in-depth explanation of each item.
- Please cite SQUIRE when it is used to write a manuscript.

Text section and item name	Page/line no(s). info is located
**Title and abstract**	
1. **Title**	1/1-2/1
Indicate that the manuscript concerns an initiative to improve healthcare (broadly defined to include the quality, safety, effectiveness, patient-centredness, timeliness, cost, efficiency and equity of healthcare).	
Manuscript concerns an initiative to improve healthcare for forcibly displaced people.	
2. **Abstract**	1/20-2/9
a. Provide adequate information to aid in searching and indexing.	1/21-2/9
b. Summarise all key information from various sections of the text using the abstract format of the intended publication or a structured summary such as: background, local problem, methods, interventions, results, conclusions.	1/21-2/9
The structure abstract summarises all key information under headings of: objective, design, setting, participants, results, and conclusions.	1/21-2/9
**Introduction: Why did you start?**	
3. **Problem description** - Nature and significance of the local problem.	2/32-3/8 and 3/28-4/11
4. **Available knowledge** - Summary of what is currently known about the problem, including relevant previous studies.	3/9-3/27
5. **Rationale** - Informal or formal frameworks, models, concepts and/or theories used to explain the problem, any reasons or assumptions that were used to develop the intervention(s) and reasons why the intervention(s) was expected to work	2/19-2/31 and 4/12-4/24
6. **Specific aims** - Purpose of the project and of this report.	4/25-4/33
**Methods: What did you do?**	
7. **Context** - Contextual elements considered important at the outset of introducing the intervention(s).	4/17-4/27

For peer review only - http://bmjopen.bmj.com/site/about/guidelines.xhtml

8. **Intervention(s)**	
a. Description of the intervention(s) in sufficient detail that others could reproduce it.	5/4-5/6 and 5/9-5/21
b. Specifics of the team involved in the work.	5/24-5/27
9. **Study of the intervention(s)**	
a. Approach chosen for assessing the impact of the intervention(s).	5/27-6/8 6/11-6/23
b. Approach used to establish whether the observed outcomes were due to the intervention(s).	6/23-6/27
10. **Measures**	
a. Measures chosen for studying processes and outcomes of the intervention(s), including rationale for choosing them, their operational definitions and their validity and reliability.	6/27-6/29
b. Description of the approach to the ongoing assessment of contextual elements that contributed to the success, failure, efficiency and cost.	7/2-7/11
c. Methods employed for assessing completeness and accuracy of data.	7/11-7/17
11. **Analysis**	
a. Qualitative and quantitative methods used to draw inferences from the data.	7/2-7/10
b. Methods for understanding variation within the data, including the effects of time as a variable.	6/15-6/17 and 7/10-7/17
12. **Ethical considerations** - Ethical aspects of implementing and studying the intervention(s) and how they were addressed, including, but not limited to, formal ethics review and potential conflict(s) of interest.	15/24-15/27
**Results: What did you find?**	
13. **Results**	
a. Initial steps of the intervention(s) and their evolution over time (eg, time-line diagram, flow chart or table), including modifications made to the intervention during the project.	7/20-8/28
b. Details of the process measures and outcomes.	8/31-12/7
c. Contextual elements that interacted with the intervention(s).	7/31-7/34 8/25-8/28 9/27-9/28
d. Observed associations between outcomes, interventions and relevant contextual elements.	7/22-7/28
e. Unintended consequences such as unexpected benefits, problems, failures or costs associated with the intervention(s).	10/32-11/3
f. Details about missing data.	12/23-12/25 14/13-14/18
**Discussion: What does it mean?**	
14. **Summary**	
a. Key findings, including relevance to the rationale and specific aims.	12/10-12/16 and 12/26-13/20
b. Particular strengths of the project.	12/16-12/25
15. **Interpretation**	
a. Nature of the association between the intervention(s) and the outcomes.	14/24-15/2
b. Comparison of results with findings from other publications.	12/26-12/28 12/31-12/34

For peer review only

	13/3-13/6<br>13/13-14/1
c. Impact of the project on people and systems.	11/18-11/20
d. Reasons for any differences between observed and anticipated outcomes, including the influence of context.	12/10-12/15
e. Costs and strategic trade-offs, including opportunity costs.	12/4-12/7
16. **Limitations**	
a. Limits to the generalisability of the work.	14/4-14/13
b. Factors that might have limited internal validity such as confounding, bias or imprecision in the design, methods, measurement or analysis.	14/13-14/18
c. Efforts made to minimise and adjust for limitations.	14/18-14/21
**Conclusions**	
a. Usefulness of the work.	14/24-14/29
b. Sustainability.	14/29-15/2
c. Potential for spread to other contexts.	15/2-15/5
d. Implications for practice and for further study in the field.	15/5-15/8
e. Suggested next steps.	15/8-15/10
**Other information**	
18. **Funding** - Sources of funding that supported this work. Role, if any, of the funding organisation in the design, implementation, interpretation and reporting.	15/19-15/21

Ogrinc G, et al. BMJ Qual Saf 2015;0:1–7. doi:10.1136/bmjqs-2015-004411
Downloaded from http://qualitysafety.bmj.com/ on January 2, 2017

For peer review only
